# CRISPR interference screens reveal growth–robustness tradeoffs in *Synechocystis* sp. PCC 6803 across growth conditions

**DOI:** 10.1093/plcell/koad208

**Published:** 2023-07-26

**Authors:** Rui Miao, Michael Jahn, Kiyan Shabestary, Gilles Peltier, Elton P Hudson

**Affiliations:** School of Engineering Sciences in Chemistry, Biotechnology and Health, Science for Life Laboratory, KTH—Royal Institute of Technology, Stockholm, SE-17165,Sweden; School of Engineering Sciences in Chemistry, Biotechnology and Health, Science for Life Laboratory, KTH—Royal Institute of Technology, Stockholm, SE-17165,Sweden; Max Planck Unit for the Science of Pathogens, 10117 Berlin,Germany; School of Engineering Sciences in Chemistry, Biotechnology and Health, Science for Life Laboratory, KTH—Royal Institute of Technology, Stockholm, SE-17165,Sweden; Department of Bioengineering and Imperial College Centre for Synthetic Biology, Imperial College London, London SW7 2AZ,UK; Aix Marseille Univ, CEA, CNRS, Institut de Biosciences et Biotechnologies Aix-Marseille, CEA Cadarache, 13108 Saint Paul-Lez-Durance,France; School of Engineering Sciences in Chemistry, Biotechnology and Health, Science for Life Laboratory, KTH—Royal Institute of Technology, Stockholm, SE-17165,Sweden

## Abstract

Barcoded mutant libraries are a powerful tool for elucidating gene function in microbes, particularly when screened in multiple growth conditions. Here, we screened a pooled CRISPR interference library of the model cyanobacterium *Synechocystis* sp. PCC 6803 in 11 bioreactor-controlled conditions, spanning multiple light regimes and carbon sources. This gene repression library contained 21,705 individual mutants with high redundancy over all open reading frames and noncoding RNAs. Comparison of the derived gene fitness scores revealed multiple instances of gene repression being beneficial in 1 condition while generally detrimental in others, particularly for genes within light harvesting and conversion, such as antennae components at high light and PSII subunits during photoheterotrophy. Suboptimal regulation of such genes likely represents a tradeoff of reduced growth speed for enhanced robustness to perturbation. The extensive data set assigns condition-specific importance to many previously unannotated genes and suggests additional functions for central metabolic enzymes. Phosphoribulokinase, glyceraldehyde-3-phosphate dehydrogenase, and the small protein CP12 were critical for mixotrophy and photoheterotrophy, which implicates the ternary complex as important for redirecting metabolic flux in these conditions in addition to inactivation of the Calvin cycle in the dark. To predict the potency of sgRNA sequences, we applied machine learning on sgRNA sequences and gene repression data, which showed the importance of C enrichment and T depletion proximal to the PAM site. Fitness data for all genes in all conditions are compiled in an interactive web application.

## Teaser

CRISPR interference screens of *Synechocystis* in different growth regimes allowed mapping of condition-specific gene fitness.

**IN A NUTSHELL**

**Background:** Cyanobacteria are photosynthetic microbes that play a crucial role in the global carbon cycle. They convert sunlight into chemical energy and produce oxygen as a byproduct. Extant cyanobacteria are also thought to share a common ancestor with the chloroplasts of plants and algae. However, nearly half of the genes in model cyanobacteria strains are not annotated with a function. This knowledge gap is a significant obstacle for fully understanding the physiology of photosynthesis, as well as for developing cyanobacteria into efficient cell factories for bioproduction.

**Question:** We aimed to map whether genes in cyanobacteria are essential and contribute to cell fitness in different growth conditions spanning a wide range of light regimes and carbon source availabilities.

**Findings:** We screened a large gene repression library consisting of more than 20,000 individual mutants in 11 different conditions. We found that certain genes showed fitness benefits in 1 condition but were generally detrimental in others, including several genes that play critical roles in mixotrophy and photoheterotrophy, indicating their significance in redirecting metabolic flux. We also discovered genes with condition-specific importance and identified additional functions for central metabolic enzymes. We compiled the fitness data for all genes in all conditions in an interactive, open-access web application. In addition, we used our extensive data set in combination with machine learning to identify “design rules” for effective guide RNAs in the cyanobacterium *Synechocystis.*

**Next steps:** Our results provide important insight to the cyanobacteria community and to plant scientists looking for cyanobacterial homologs to chloroplast proteins or other examples of suboptimality in regulation of photoprotection. Future studies may focus on investigating the highlighted genes or regulatory pathways with individual mutants.

## Introduction

Photoautotrophic microbes, including various cyanobacterial strains, have been pursued as next-generation catalysts for synthesis of fuels or chemicals (Lips et al. 2018). In addition to their biotechnological potential, cyanobacteria have served as model organisms for photosynthesis and carbon fixation, due to an ancestral relationship to the chloroplasts of photosynthetic eukaryotes. Nevertheless, 45% of genes in the most-studied cyanobacterium *Synechocystis* sp. PCC 6803 are not annotated with a function, compared with 15% to 35% in the model heterotroph *Escherichia coli* (Ghatak et al. 2019). This knowledge gap is a significant obstacle to fully understanding cyanobacterial physiology, as well as to developing cyanobacteria into efficient cell factories for bioproduction. In recent years, there have been a number of technical advances that accelerate high-throughput functional genomics. Barcoded mutant libraries, created via transposon, CRISPR/Cas, or Clustered Regularly Interspaced Short Palindromic Repeats Interference (CRISPRi), allow mutant tracking via NGS and thus the screening of thousands of genes simultaneously across environmental conditions (Garst et al. 2017; Price et al. 2018; Wang et al. 2018; Yao et al. 2020; Jahn et al. 2021; Vo et al. 2021). Such a highly parallel experimental format facilitates identification of condition-specific gene fitness.

Over the course of evolution, microorganisms have acquired the genetic inventory and regulatory mechanisms to provide quick responses toward dynamic environments. For example, the lithoautotroph *Cupriavidus necator* simultaneously expresses multiple alternative pathways for substrate assimilation (Jahn et al. 2021), and *Pseudomonas putida* precautionarily expresses numerous efflux pumps to increase solvent and xenobiotic tolerance (Ramos et al. 2015). A systems biology analysis suggested that *Synechocystis* does not efficiently regulate proteins involved in light harvesting and CO_2_ fixation in high-growth conditions, possibly retaining excess enzyme capacity in anticipation of changing conditions (Jahn et al. 2018), but the same expression can be burdensome for growth in another environment. Robustness in the presence of light fluctuations is a well-studied phenomenon in photosynthetic organisms. In plants, several nonphotochemical quenching (NPQ) routes are sustained when conditions are no longer stressful due to low expression of key enzymes, so that rapid deactivation through additional enzyme expression is an objective for increasing crop yield (Kromdijk et al. 2016). Again, the high parallelization of barcoded library functional genomics allows the study of possible suboptimal, or “wasteful,” gene expression across the genome and across multiple conditions.

Here, we have used a pooled CRISPR interference library to elucidate the contribution of *Synechocystis* genes to cell growth in specific conditions and discover fitness tradeoffs that highlight evolutionary pressures to adapt to changing environments. A previous CRISPRi library for *Synechocystis* contained 2 single guide RNAs (sgRNAs) targeting each gene, which resulted in ambiguous results when 1 sgRNA gave a growth phenotype and the other did not (Yao et al. 2020). Our expanded CRISPRi library has the majority of genes targeted by 5 sgRNAs to increase confidence in derived fitness scores, as well as sgRNAs targeting noncoding RNAs (sRNAs, antisense RNAs [asRNAs], and alternative transcription start sites), a widespread class of potentially regulatory molecules in cyanobacteria (Kopf and Hess 2015). The expanded CRISPRi library was cultivated in 11 different conditions where carbon source, nitrogen source, and light availability varied. The resulting large data sets reveal previously unannotated genes as being important for cell growth in certain conditions, as well as several examples of growth–robustness tradeoffs, where gene repression accelerated growth in 1 condition but was detrimental in others. All analyzed fitness data for competition experiments can be accessed on an interactive web application (https://m-jahn.shinyapps.io/ShinyLib/).

## Results

### An expanded CRISPRi library for the cyanobacterium *Synechocystis* sp. PCC 6803

**Figure 1.**
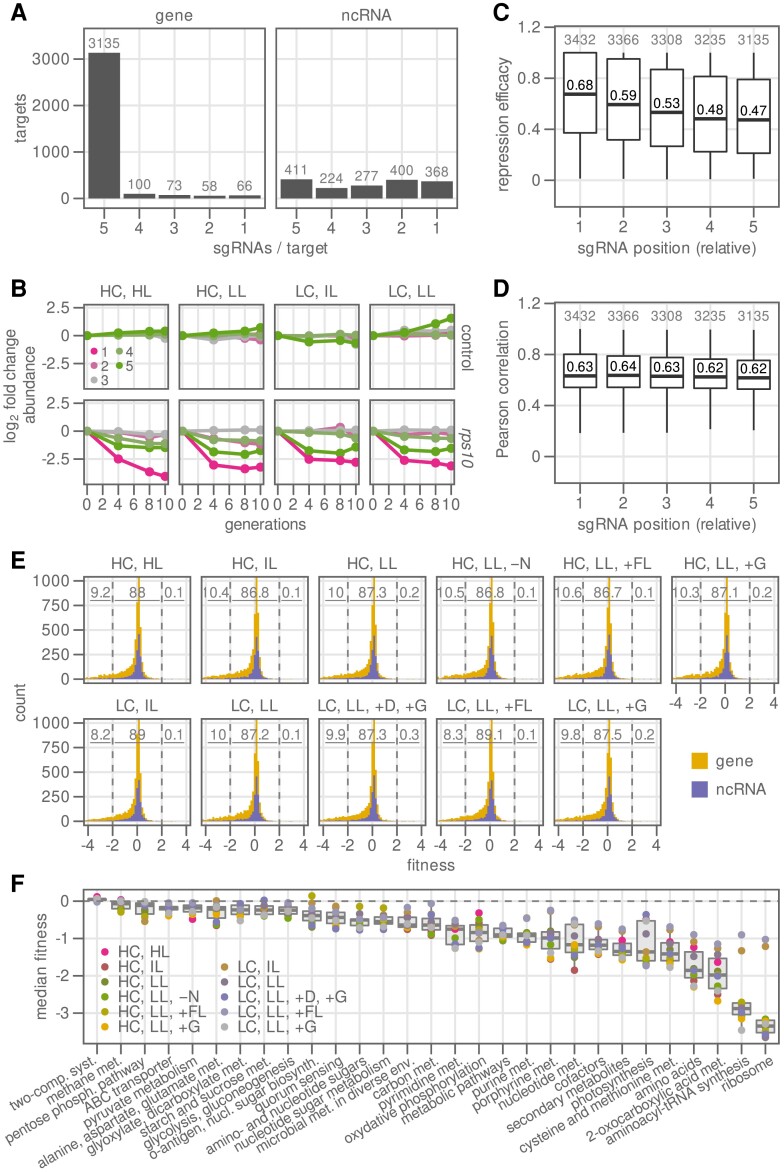
*Synechocystis* CRISPRi library targets most genes with 5 sgRNAs. A CRISPRi repression library was constructed for *Synechocystis* sp. PCC 6803 targeting 3,432 genes and 1,712 ncRNAs. **A)** Almost all genes (3,136 of 3,432, 91%) were targeted with 5 single guide RNAs (sgRNAs), while ncRNAs were often targeted with fewer sgRNAs because of limited sequence length. **B)** Abundance log_2_ fold change of example mutants over a time course from 0 to 10 generations in 4 different conditions. For full names of conditions, see Table 1. Upper row, 5 nontargeting sgRNA controls; lower row, 5 sgRNAs targeting the ribosomal gene *rps10*. Numbering is relative distance of sgRNA to start codon (1, closest; 5, most distant). **C)** Relative repression efficacy of an sgRNA depending on distance to the start codon (1, closest; 5, most distant). The sgRNA with the strongest effect on fitness is set to a value of 1. Only sgRNAs targeting genes were included for the analysis. Box limits, 25th and 75th percentile; central line, median; and whiskers, 1.5-fold interquartile range. **D)** Pearson’s correlation coefficient of each sgRNA to the other sgRNAs targeting the same gene, depending on distance to the promoter. The correlation coefficient was rescaled to a range of 0 to 1. The same set of sgRNAs and the same type of box plot as in **C)** was used. **E)** Histogram of fitness score per condition, for genes (yellow) and ncRNAs (purple). Inset numbers show the percentage of genes falling in 3 different bins: strong negative fitness (−4 to −2), no or weak effect on fitness (−2 to 2), and strong positive fitness (2 to 4). For full names of conditions, see Table 1. **F)** Median fitness score of genes per pathway (KEGG), broken down by cultivation condition (*n* = 11; see Table 1). Box plots are defined as in **C)**.

The CRISPRi library was constructed so that both the catalytically inactive Cas9 enzyme (dCas9) and sgRNA are transcribed by anhydrotetracycline (aTc)-inducible promoters. Upon addition of aTc, the dCas9 and sgRNA are expressed, and the dCas9-sgRNA complex mediates blockage of transcription, with gene specificity determined by the sgRNA sequence (Qi et al. 2013; Yao et al. 2016, 2020). Up to 5 sgRNAs were designed to target each gene and noncoding RNA (ncRNA) on the chromosome and the native plasmids of *Synechocystis* (Supplemental Data Set 1). Five sgRNAs were designed for most genes (92%), while fewer sgRNAs were generated for shorter open reading frames (ORFs) and for ncRNAs (25% of ncRNAs were targeted by 5 sgRNAs) (Fig. 1A). The resulting 21,705 sgRNAs were synthesized as a pool (GenScript) and integrated into a *Synechocystis* strain harboring dCas9. All transformants, where each cell contained a single sgRNA, were scraped off agar plates, and this constituted the pooled CRISPRi library (Materials and methods). The presence of all designed sgRNAs in the pooled library was verified by next-generation sequencing (NGS).

We performed turbidostat cultivations of the pooled library with varying CO_2_ concentration, light exposure, and additional treatments such as the addition of glucose to enable mixotrophic growth, the addition of glucose and DCMU for photoheterotrophic growth, and nitrogen starvation (Table 1). Conditions were expected to put demands on carbon and energy metabolism, which cyanobacteria must balance to maximize growth rate. The composition of the pool in each condition was determined by NGS of sgRNA cassettes after 0, 4, 8, and 10 generations. The change in abundance of each library member over the cultivation duration is a measure of the relative growth rate of that member and was used to determine a fitness score (Love Huber, and Anders 2014; Yao et al. 2020) (Fig. 1B). Each sgRNA for a gene may have a different efficacy in vivo and thus different magnitude of effect on cell growth (fitness score). We found that sgRNAs targeting most proximal to the promoter of the target gene (“position 1”) had the highest absolute fitness scores and thus presumably higher repression efficacy (median = 0.68, where relative fitness is scaled between 0 and 1; Fig. 1C). The absolute fitness score of sgRNAs declined with distance from the promoter (position 5, median = 0.47), which is similar to previous reports (Wang et al. 2018).

**Table 1.** Summary of the conditions used in turbidostat cultivation of the pooled library

Condition ID	Condition	CO_2_ supplement	Light intensity (*µ*mol photons m^−2^ s^−1^)	Generation time (h)
HC and HL	High CO_2_, high light	1%	1,000	15
HC and IL	High CO_2_, intermediate light	1%	150	13
HC and LL	High CO_2_, low light	1%	60	26
HC, LL, and −N	High CO_2_, low light, N**−** limitation	1%	60	27
HC, LL, and +FL	High CO_2_, low light, light pulses	1%	Constant 60 + 5 s 1,500 every 5 min	23
HC, LL, and +G	High CO_2_, low light, glucose	1%	60	18
LC and IL	Low CO_2_, intermediate light	0.04%	150	50
LC and LL	Low CO_2_, low light	0.04%	60	29
LC, LL, and +FL	Low CO_2_, low light, light pulses	0.04%	Constant 60 + 5 s 1,500 every 5 min	69
LC, LL, and +G	Low CO_2_, low light, glucose	0.04%	60	14
LC, LL, +D, and +G	Low CO_2_, low light, glucose, DCMU	0.04%	60	21

Constant light was supplied by the white light LED on the back of the bioreactor (Multi-Cultivator MC-1000-OD bioreactors (Photon System Instruments, Drasov, CZ). Fluctuating light (1,500 *µ*mol photons m^−2^ s^−1^) was provided by an extra LED light panel, PARADIGM LIGHT WH 1200-V (Beambio), from the front side of the photobioreactor.

We also confirmed that the position of an sgRNA has no effect on the correlation with other sgRNAs for the same gene (Fig. 1D). Gene fitness scores were calculated from individual sgRNA fitness scores by weighted mean, and significance was determined by calculating the multiple-hypothesis–adjusted *P* value (*P*_adj_) from individual sgRNA fitness scores of the same target (Materials and methods). The results of the library competition experiments are available on the interactive R Shiny–based web application ShinyLib (https://m-jahn.shinyapps.io/ShinyLib/). Users can select their genes and conditions of interest and visualize fitness score, sgRNAs fold change over time, and other metrics using dot plots and heat maps.

The majority of targets that showed an effect on fitness were genes (Fig. 1E), while only few ncRNAs showed an independent effect upon repression. To obtain an overview of the metabolic pathways most important for cell fitness, genes were sorted by Kyoto Encyclopedia of Genes and Genomes (KEGG) pathways and the median fitness per pathway in each condition was calculated (Fig. 1F). As expected, most genes that exhibited an impact on fitness demonstrated a decrease in fitness, leading to negative median fitness scores across various conditions for most pathways. The metabolic pathways showing the strongest detrimental effect on fitness upon repression were associated with ribosomes, nucleotide and tRNA biosynthesis, and amino acid biosynthesis. Noncentral dogma pathways with strong average fitness loss included photosynthesis, biosynthesis of cofactors and secondary metabolites including chlorophyll, and oxidative phosphorylation. Interestingly, all of these pathways were associated with energy metabolism, while the carbon metabolic pathways showed milder effects, with first appearances for central carbon metabolism and glycolysis and gluconeogenesis at rank 15 and 21, respectively. This relatively weak fitness effect could be due to high enzyme abundance in central carbon metabolism (underutilization of enzymes), a higher fraction of isoenzymes than in energy metabolism, or compensation by redirection of metabolic flux through other pathways.

In 2 cultivation conditions, low CO_2_ with intermediate light (LC and IL) and low CO_2_ with fluctuating light (LC, LL, and +FL), the fitness penalty for repression of ribosomal and tRNA biosynthesis genes was substantially weaker than in other conditions, while other essential genes, such as those related to photosynthesis, had fitness penalties similar to other conditions (Fig. 1F). Cells in these conditions were under extreme light stress and had low growth rate, so that the demand for active ribosomes was reduced. Additionally, bacteria keep significant reserves of inactive ribosomes at low growth, a phenomenon explored in detail mostly in *E. coli* but also recently inferred in cyanobacteria (Dai et al. 2016; Jahn et al. 2018; Wu et al. 2022). A ribosome profiling study of *Synechocystis* estimated that 40% of ribosomes were in an inactive 100S state in an LC and IL condition, compared with 20% in a HC and IL condition (Karlsen et al. 2018). Extremely low growth rate and significant ribosomal reserves may result in a relative insensitivity to ribosome repression by CRISPRi in the LC and IL condition.

In order to identify genes particularly important for fitness in changing carbon or light conditions, but not both, we applied multiple linear regression modeling to our fitness data. These models can dissect the influence of the single variables carbon concentration, light intensity, and additional treatments on the fitness score of each gene. We selected 187 genes with significantly changed fitness in any condition (threshold: 4.0), clustered these using t-SNE, and overlaid the results from multiple linear regression (Supplemental Fig. S1). We found several genes (and gene clusters) of interest which we analyze in detail in the following sections, for example, photosystem and phycobilisome (PBS) subunits (*psb* and *apc*) in mixo- and photoheterotrophic conditions, flavodiiron (Flv) proteins (*sll0217* and *sll0219*) in C_i_-limited conditions, and the proposed CBB cycle regulator CP12 (*ssl3364*).

### Fitness tradeoffs for anticipating light stress and carbon limitation

**Figure 2.**
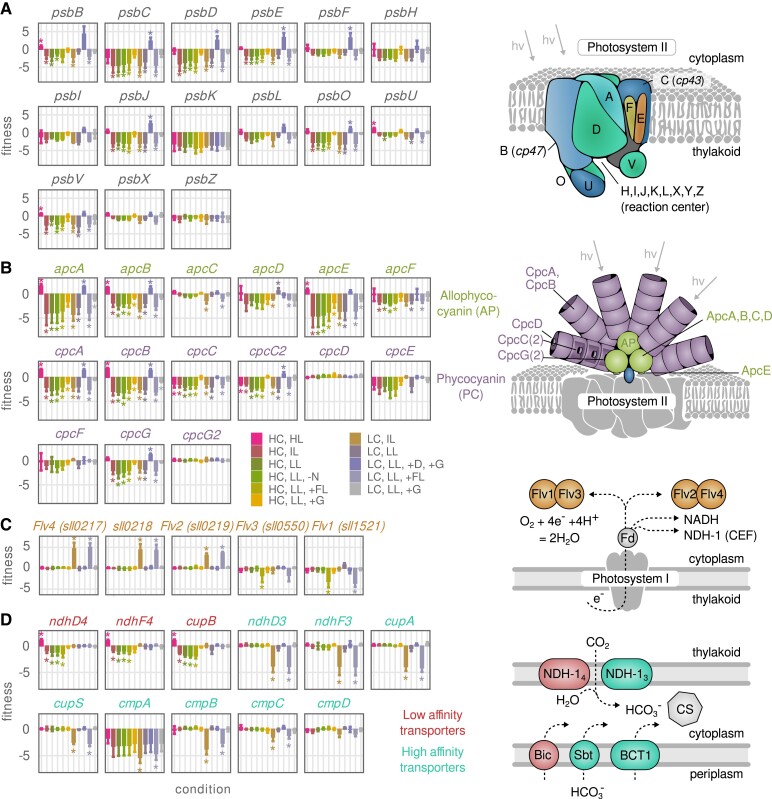
Adaptations to excess carbon or light are suboptimal in some environments. **A)** Fitness score for repression of selected genes encoding photosystem II subunits. Fitness scores for all photosystem I and II subunits can be found in Supplemental Fig. S1. Asterisk: Wilcoxon rank sum test–adjusted *P* value ≤ 0.01. Error bars represent weighted mean and Sd of up to 5 sgRNAs, each quantified with 4 biological replicates. Illustration: photosystem II structure adapted from KEGG (Kanehisa et al. 2017). hv, photon energy. For full names of conditions, see Table 1. **B)** Fitness score for repression of genes encoding PBS subunits. Illustration: PBS structure adapted from KEGG. **C)** Fitness score for repression of genes encoding Flv proteins. Illustration: reported electron flow from PSI to Flv proteins. Fd, ferredoxin. CEF, cyclic electron flow. **D)** Fitness score for repressing genes encoding selected carbon transporters in *Synechocystis*: NDH-1_4_ complex (*ndhD4*, *ndhF4*, and *cupB*), NDH-1_3_ complex (*ndhD3*, *ndhF3*, *cupA*, and *cupS*), and BCT1 (*cmpABCD*). Red, low-affinity C_i_ transporters; turquoise, high-affinity C_i_ transporters. Illustration: location of different Ci transporters and corresponding forms of Ci they transport. CS, carboxysome.

Repression of the PSI subunits A, B, C, and D was detrimental for growth in all conditions (Supplemental Fig. S2), which is in agreement with their integral role in PSI formation and function (Yu et al. 1995; Malavath et al. 2018). Repression of the small PSI subunits E, F, J, K, L, I, and M also affected growth negatively, but in most conditions, the effect was weak (Supplemental Fig. S2). This is congruent with previous reports that the small subunits could be deleted in *Synechocystis* without effect on growth (Jeanjean et al. 2008; Malavath et al. 2018). Slightly stronger negative effects on growth were observed in conditions with low CO_2_ with intermediate or fluctuating light (LC and IL and LC and FL), which may indicate that the small PSI subunits may play a role in photoprotection under light stress conditions. Repression of genes encoding PSII reaction center proteins *psbC*, D2 (*psbD*), and *psbK* resulted in strong negative fitness effects (Fig. 2A).

The D1 subunit of the reaction center is encoded by 3 genes, of which the *psbA1* encoded D1 variant is specific to low-oxygen conditions (not tested in this study) (Summerfield et al. 2008; Crawford 2016). The *psbA1* locus is often considered a neutral site (Mohamed and Jansson 1989) and was used here to integrate the dCas9 gene construct. The repression of either of the 2 other copies, *psbA2* and *psbA3*, did not result in significant fitness change in any tested conditions, suggesting these genes can compensate for each other. When cells were grown on glucose in the presence of DCMU to block the electron transfer from PSII to plastoquinone (photoheterotrophy), most PSII genes showed growth advantage upon repression. This could be a result of a combined effect of reduced protein burden and reduced oxidative stress. Since photosynthesis is not required in this condition, silencing of unused proteins may allow cells to reinvest resources into other functions (Jahn et al. 2018, 2021). When DCMU is added to cells under illumination, the blocked electrons elevate reactive oxygen species (ROS) production at PSII. Therefore, repressing PSII expression might reduce the possibility of high ROS accumulation, which could further benefit cell growth. However, PsbK was an exception, as it was also critical for cell growth even in photoheterotrophic condition. Therefore, while other PSII subunits appear to be dispensable when glucose is present (Ikeuchi et al. 1991), PsbK may have a universal role in electron transport which is not restricted to PSII function.

The apparent wasteful expression of PSII in photoheterotrophic conditions motivated us to look for other instances of potential suboptimal regulation, particularly in light harvesting and conversion, where preparedness for a potential shift in light intensity could come at the expense of growth rate. One example is the expression of the PBS, a large membrane-extrinsic protein complex serving as a photon-capturing antenna (Adir et al. 2020). PBS is a significant proportion of the cellular proteome (20% to 40% by mass; Supplemental Fig. S3) (Grossman et al. 1993; Jahn et al. 2018), and the expression of PBS genes is regulated in response to perceived light, with expression increasing at low light or in presence of DCMU and decreasing in high light (MacKenzie et al. 2005; Jahn et al. 2018; Zavřel et al. 2019).

However, the cyanobacterium *Synechocystis* does not fully repress PBS even at extreme light, though photosynthesis can still occur when the entire PBS is knocked out (Ajlani et al. 1995). Instead, excessive PBS excitation is quenched in high light by the orange carotenoid protein (OCP) (Domínguez-Martín et al. 2022). Additionally, PBS may partially detach from PSII to prevent energy transfer (Calzadilla and Kirilovsky 2020). Several studies have proposed artificial antennae truncation to reduce the effective cross-section for light capture and thus prevent wasteful NPQ in cyanobacteria cultures (Grossman et al. 1993; Melis 2009; Kirst et al. 2014; Lea-Smith et al. 2014). The CRISPRi fitness data showed a general negative effect of PBS repression across most conditions, except for extreme light (HC and HL) and photoheterotrophy (LC, LL, +G, and +D) conditions, where repression of ApcAB and CpcAB clearly improved growth rate (Figs. 2B and S3). The magnitude of the growth defect of PBS repression was also reduced in the mixotrophy (+G) conditions. Thus, in most conditions, repression of the antennae is detrimental, though in conditions where the antennae are not needed, repression led to an increase in growth rate (see Discussion for possible explanations).

A second example of a condition-specific tradeoff between robustness and growth rate was observed for the Flv proteins. Flvs protect cyanobacteria from excess light energy and thus overreduction of NAD(*P*) by reducing molecular oxygen to water (Fig. 2C) (Allahverdiyeva et al. 2013). In *Synechocystis*, 4 Flv proteins are known (Flv1 to 4), of which Flv1/3 and Flv2/4 associate as heterodimers. *Flv1/3* is constitutively expressed, essential for survival on intense fluctuating light, and caps excess light energy at both low- and high-carbon levels (Santana-Sanchez et al. 2019). In agreement to this, we observed a significantly reduced growth of *flv1* and *flv3* repression clones in fluctuating light (+FL) conditions regardless of CO_2_ concentration (Fig. 2C). Surprisingly, *flv2* and *flv4* repression clones had fitness increases that were specific to the 2 low C_i_ and excess light conditions (LC and IL and LC, LL, and +FL). Flv2 and Flv4 were previously shown to be involved in dissipating electrons as a photoprotection mechanism (Santana-Sanchez et al. 2019). An increased fitness in *flv2* and *flv4* knockdowns could potentially arise from a better protein economy or electron economy.

Flv2 and Flv4 are significantly upregulated in low C_i_ conditions and are responsible for O_2_ reduction at steady state (Zhang et al. 2009; Santana-Sanchez et al. 2019; Nikkanen et al. 2020). In the LC, LL, and +FL condition, Flv2/4 may not have a beneficial role as most of the electrons are produced in a fast and transient manner, which are dissipated by Flv1/3. Therefore, repressing *flv2* or *flv4* in this condition may save protein resources or prevent wasteful O_2_ reduction. In LC and IL condition, cells have constant light stress, and a form of NAD(P)H dehydrogenase-like complex (NDH-1_3_) is also upregulated, presumably to accelerate electron dissipation and C_i_ assimilation (Santana-Sanchez et al. 2019; Zhang et al. 2020). In contrast to *flv2* and *flv4* repression, repression of *ndh* subunits under LC and IL and LC, LL, and +FL had a significant fitness penalty. Moreover, when Flv2/4 is knocked out, the expression of Flv3 is dramatically increased, and Flv1/3 was shown to be more efficient than Flv2/4 at oxygen uptake (Santana-Sanchez et al. 2019). We also found that deletion of Flv2 and Flv4 does not change the photosynthetic parameter Fv/Fm (a reporter of PSII quantum yield) in the absence of bicarbonate (Supplemental Fig. S4C). In addition, we observed slightly higher oxygen production rate in ΔFlv2 mutant compared with WT, though its oxygen uptake capacity remained low (Supplemental Fig. S4, A and B). Therefore, we hypothesize that in these 2 specific conditions, the repression of Flv2 (or Flv2/4) saves protein resources, while upregulation of NDH-1_3_ and Flv3 is sufficient to dissipate excess electron flow. On the other hand, Flv2/4 mutants did not show any change on growth in the LC and LL condition, because NDH-1_3_ expression is not upregulated and there are no excess electrons that need to be dissipated.

Another potential tradeoff was observed on the repression of Ci transporter NDH-1_4_ in HC and HL condition. *Synechocystis* possesses 5 major C_i_ transporters (reviewed in (Price 2011)), among which are 2 low-affinity/high-flux transporters (sodium-dependent bicarbonate transporters [BicA and NDH-1_4_]) and 3 high-affinity/low-flux transporters (sodium-dependent bicarbonate transporter [SbtAB], the bicarbonate transporter BCT1, and NDH-1_3_). NDH-1_4_ is constitutively expressed for basal C_i_ uptake, while the expression of the other C_i_ transporters is induced in C_i_-limited conditions (Shibata et al. 2001; Zhang et al. 2004). CRISPRi repression of the NDH-1_4_ subunits reduced growth in most high-carbon conditions and had no effect in low-carbon conditions (Fig. 2D), indicating that NDH-1_4_ is the major C_i_ transporter when CO_2_ is abundant. However, repression of NDH-1_4_ increased growth in the high-carbon and high-light condition (HC and HL). A plausible explanation could be that NDH-1_4_ becomes a protein burden when NDH-1_3_ is upregulated in high light in order to increase cyclic electron flow, and it takes over a part of the CO_2_ transport (Bernát et al. 2011), (Zhang et al. 2020).

In C_i_-limiting conditions, high-affinity Ci transporters are essential for efficient carbon uptake; thus, NDH-1_3_ (*ndhD3*, *ndhF3*, *cupA*, and *cupS*) and BCT1 (*cmpABC*) repressing mutants showed significantly reduced fitness in 2 conditions characterized by a high ratio of light over CO_2_ supply (LC and IL and LC, LL, and +FL) (Fig. 2D). We also examined genes involved in the C_i_ regulatory network. Repression of low-affinity C_i_ transporter inhibitor *ccmR* (also named *ndhR* and *rbcR*) increased cell fitness in the LC and IL condition, and being consistent with previous studies, the repression of *cyabrB1* is lethal and the repression of *cyabrB2* reduced cell fitness in all conditions (Ishii and Hihara 2008; Kaniya et al. 2013; Orf et al. 2016) (Supplemental Fig. S5).

### Essentiality of genes in central carbon metabolism

**Figure 3.**
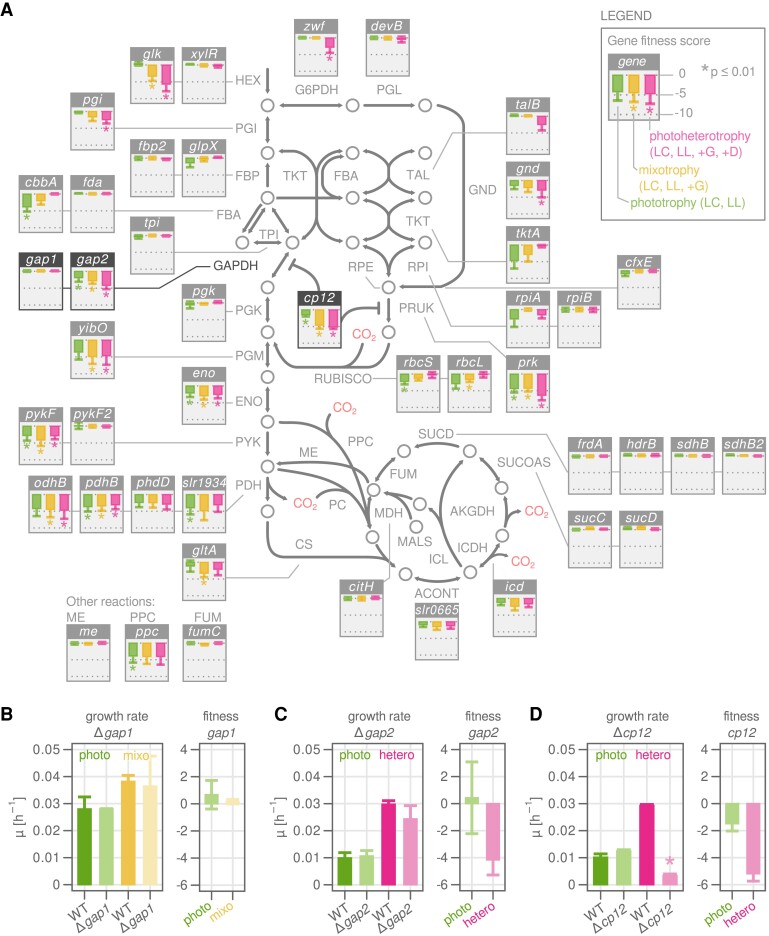
Essentiality of genes in central carbon metabolism. **A)** Metabolic map of central carbon metabolism for *Synechocystis sp*. PCC 6803. Reactions and their directionality are shown with arrows and named with capital letters according to the BiGG standard. For full reaction names, see Supplemental Table S3. Metabolites are shown as circles. Minifigures show fitness scores for the genes associated with the respective metabolic reaction. Green bars, phototrophy (“photo”); yellow bars, mixotrophy (“mixo”); and red bars, photoheterotrophy (“hetero”). Error bars represent weighted mean and Sd of up to 5 sgRNAs, each quantified with 4 biological replicates. Asterisk: *P* value from Wilcoxon rank sum test lower than 0.01. For full names of conditions, see Table 1. **B)** Validation of CRISPRi library results for Gap1 using a Δ*gap1* deletion strain. Cultivation was performed in the same conditions as were used for cultivating the CRISPRi library, but in batch mode instead of turbidostat. Detailed growth curves are shown in Supplemental Fig. S8. Phototrophy: HC and LL. Mixotrophy: HC, LL, and +G. The WT strain was used as control in each cultivation condition, and the specific growth rate *μ* was calculated as the slope of log OD_720_ between hour 20 and 40. The fitness score of the corresponding gene is shown for comparison. Error bars: mean and Sd of at least 2 biological replicates. Asterisk: *P* value from Student’s *t* test lower than 0.01. **C)** As in **B)** but for *gap*2. Phototrophy: LC and IL. Photoheterotrophy: LC, LL, +G, and +D. **D)** As in **B)** but for *cp12*. Phototrophy: LC and LL. Photoheterotrophy: LC, LL, +G, and +D.

*Synechocystis*, like many other bacteria, has gene duplications or isoenzymes that can theoretically compensate for the loss or repression of a central carbon metabolism gene. Although the metabolic flux through most major reactions of central metabolism is known (Nakajima et al. 2014; You et al. 2015), it is often not easy to determine which genes/isoenzymes contribute most to carrying a reaction’s flux. We compared the fitness of central carbon metabolism genes in 3 growth conditions, phototrophy, mixotrophy, and photoheterotrophy (Fig. 3A). An essential reaction catalyzed by a single enzyme will lead to a strong fitness penalty when the corresponding gene is repressed. On the other hand, a reaction catalyzed by 2 isoenzymes will show either a partial fitness penalty (reduced flux) or no penalty at all (unchanged flux) if 1 enzyme can compensate for the loss of the other. In cases where multiple genes are annotated for a reaction, comparison of fitness scores can reveal that 1 gene is more important than another.

For example, we confirmed that *glk* (*sll0593*) but not *xylR* (*slr0329*) encodes the main hexokinase (HEX) for glucose import (Fig. 3A), which is consistent with a previous study comparing *glk/xylR* knockouts (Lee et al. 2005). Similarly, *cbbA* (*sll0018*) but not *fda* (*slr0943*) was identified to be responsible for 90% of total fructose bisphosphate aldolase (FBA) activity (Nakahara et al. 2003), and we indeed observed reduced fitness scores in all photoautotrophic and mixotrophic conditions for *cbbA* repression (Fig. 3A). Furthermore, our results indicate that 1 of the pyruvate kinase (PYK) isoforms encoded by *pykF* (*sll1275*) is more important in all conditions than the isoform encoded by *pykF2* (*sll0587*) (Fig. 3A), which was predicted to be highly inhibited by ATP in an in silico study (Haghighi 2021). In general, gene fitness scores corresponded well with expected enzyme usage based on reported fluxes (Supplemental Fig. S6) (Nakajima et al. 2014). A high fitness penalty for the repression of the oxidative PPP (G6PDH, PGL, and GND) was evident for photoheterotrophic growth, but not in the other conditions. The TCA cycle on the other hand carried very low flux in all conditions (Supplemental Fig. S6), and accordingly, repression of most TCA associated genes had nearly no effect on fitness (Fig. 3A).

It is important to keep in mind that enzyme abundance is usually higher than what is minimally required to maintain flux. Such a “reserve flux capacity” was shown to increase robustness to perturbations or environmental changes in different bacteria (O'Brien et al. 2016; Mori et al. 2017; Christodoulou et al. 2018; Sander et al. 2019). As a consequence, incomplete repression can leave sufficient residual enzyme capacity to maintain the flux of a reaction, which may complicate the interpretation of fitness scores. An example of this is Rubisco, which showed moderately but not dramatically reduced fitness for both subunits (*rbcS* and *rbcL*), despite being the primary source of carbon fixation during photoautotrophic growth. Another example is phosphoribulokinase (Prk), the committed step for CO_2_ fixation that provides the precursor ribulose-1,5-bisphosphate for Rubisco. Surprisingly, the growth penalty for Prk repression was highest for photoheterotrophy (Fig. 3A), a condition where flux through the enzyme has been shown to be low (Supplemental Fig. S6). The relative importance of Prk in photoheterotrophic conditions may be due to its role in the ternary complex Prk-CP12-Gap2.

We also found some discrepancies among the reported fluxes and calculated fitness scores for the associated enzymes. Phosphoglycerate kinase (Pgk) and glyceraldehyde 3-phosphate dehydrogenase (GAPDH) connect the important branch point metabolite 3-phosphoglycerate with the upper part of glycolysis and the CBB cycle; the reactions carry high flux during mixotrophic growth, but not during photoheterotrophic growth (Supplemental Fig. S6) (Nakajima et al. 2014; You et al 2015). However, we found that the fitness penalty for 1 of the GAPDH isoenzymes, Gap2, was highest during photoheterotrophic growth, while Gap1 and Pgk repression had no penalty (Fig. 3A). Of the 2 GAPDH isoenzymes in *Synechocystis*, *gap2* (*sll1342*) has been intensively studied (Valverde et al. 1997), including its redox-dependent association with the small regulatory protein CP12 (*ssl3364*) and Prk (Michelet et al. 2013; McFarlane et al. 2019; Lucius et al. 2022), and is known to participate in both gluconeogenesis and glycolysis (Valverde et al. 1997). In contrast, little is known of the physiological role of Gap1 (*slr0884*). Repression of *gap1* showed no fitness effect in any tested condition (Fig. 3A), which is in contrast to a previous study in which a *gap1* mutant could not grow in a mixotrophic condition (Koksharova et al. 1998). We also found that repression of *cp12* resulted in a similar growth pattern to *gap2*: significantly reduced growth in mixotrophic and photoheterotrophic conditions. This suggests that CP12 plays a role in regulating the Calvin cycle in the presence of glucose, as recently reported (Blanc-Garin et al. 2022; Lucius et al. 2022).

To validate a specific gene from the CRISPRi library results, individual knockdown or knockout clones need to be created, since the library was generated as a pool. We constructed several in-frame knockout mutants, including Δ*gap1*, Δ*gap2*, and Δ*cp12* (Figs. 3, B to D, and S7), by integrating a chloramphenicol resistance gene. Because *Synechocystis* has multiple copies of its chromosome and megaplasmids, segregation of all chromosome copies were checked and we could not obtain a fully segregated Δ*gap2* strain, while complete *gap1* and *cp12* knockouts were possible. The Δ*gap1*, Δ*gap2* (partial), and Δ*cp12* strains had phenotypes similar to the repression clones from the CRISPR library; *gap1* knockout did not affect growth in photoautotrophic or mixotrophic conditions, while *gap2* and *cp12* knockouts reduced growth in the presence of glucose (Figs. 3, B to D, and S8). It has been shown that the Gap2 of *Synechococcus* can accept NAD(H) or NADP(H) cofactors, but this preference shifts toward NAD(H) upon binding to CP12 and further when the ternary Gap2-CP12-Prk complex is formed, as NADP(H) activity is reduced to zero (McFarlane et al. 2019). Binding of Gap2 and CP12 (and potentially Prk) in dark conditions may thus regulate the change in metabolic flux from gluconeogenesis to glycolysis direction. Our results are further evidence for the importance of a fine-tuned regulation on Gap2 for both inorganic carbon uptake by the Calvin cycle and organic carbon assimilation by glycolysis.

### Fitness effects of ncRNAs across growth conditions

The CRISPRi repression library also contains 4,950 mutants with sgRNAs targeting 1,868 ncRNAs. Of these, 85% were alternative transcriptional units directly associated with a gene such as asRNAs or internal transcription start sites (iTSS) (Table 2). Small RNAs (sRNAs) were 15% of the targeted ncRNAs. sRNAs are presumably independent transcriptional units located between annotated ORFs, and several have been implicated in regulating gene expression (Mitschke et al. 2011; Kopf and Hess 2015). Only a few ncRNAs showed an effect on fitness (Table 2). Of the different ncRNA classes, iTSS showed the highest proportion of elements with significant effect on fitness and asRNAs the lowest (Table 2 and Supplemental Fig. S9A). This result is not surprising; sgRNAs targeting iTSS will also repress transcription of the coding gene, but with lower efficiency.

**Table 2.** Summary of ncRNA repression effect in *Synechocystis*

ncRNA type	No. of targets	No. of targets with sign. fitness effect (score ≥ 4)	% sign. targets
asRNA	912	18	1.97
iTSS	529	61	11.53
sRNA	245	27	11.02

Significance was defined as exceeding a combined score of effect size (fitness) times negative log_10_*P* value (Wilcoxon rank sum test). Number of significant ncRNAs was determined by counting all ncRNAs that had a combined score ≥4 in at least 1 out of 11 growth conditions.

**Figure 4.**
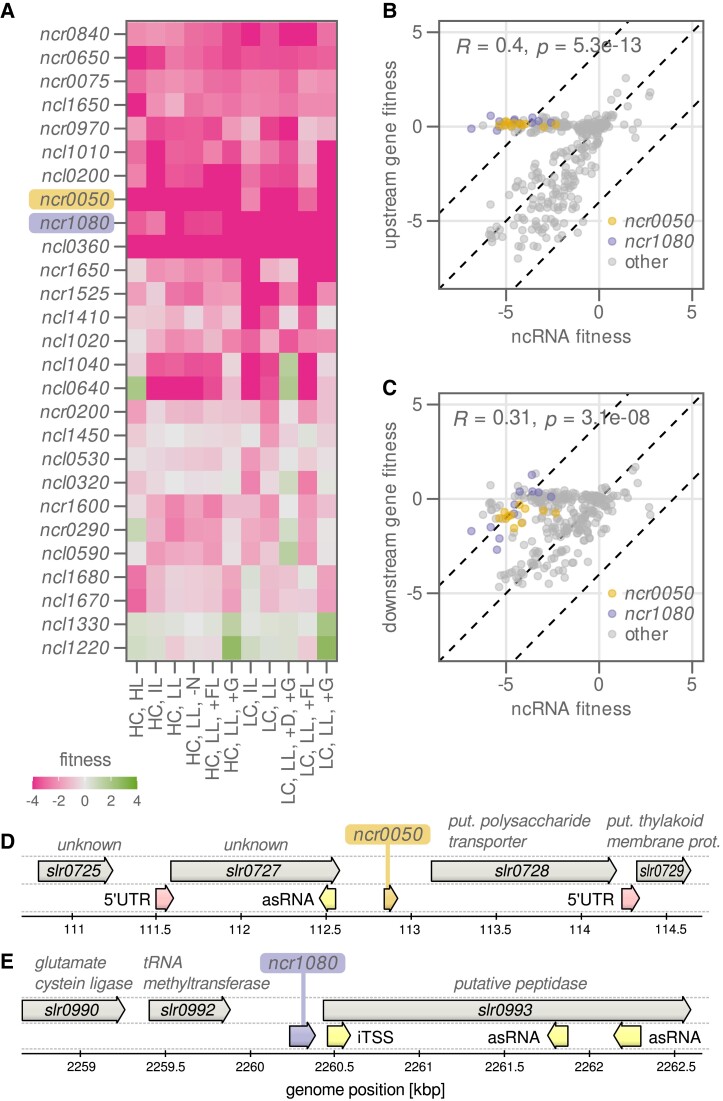
Most ncRNAs do not have an independent effect on fitness in the tested conditions. **A)** Heat map showing fitness score of selected sRNAs in 11 different growth conditions. For full names of conditions, see Table 1. Gene fitness score was determined from up to 5 sgRNAs, each quantified with 4 biological replicates. **B)** Correlation of fitness score of the 27 sRNAs in **A)** with fitness score of their respective upstream located genes. Every dot represents 1 sRNA in 1 growth condition. R, correlation coefficient. *P*, probability that coefficient is zero. **C)** As in **B)** but for the downstream located genes. Colored dots highlight ncRNAs with position independent fitness effects. **D)** Genomic context of the sRNA *ncr0050*. 5′ UTR, 5′-untranslated region. asRNA, antisense RNA. iTSS, internal transcription start site. kbp, kilo base pairs. **E)** Genomic context of the sRNA *ncr1080*.

To evaluate how much of the fitness effect of asRNAs and iTSS repression was actually caused by repression of the associated genes, we selected all ncRNAs with a significant effect on fitness in at least 1 condition and correlated this fitness with gene fitness. The correlation of fitness scores between a gene and its corresponding asRNA was high (*R* = 0.67), while correlation of fitness between a gene and iTSS was lower (*R* = 0.37) (Supplemental Fig. S9, B and C). Only a few ncRNAs were found that affected cell fitness independent of their associated gene. We therefore focused our analysis on sRNAs and found 27 sRNAs with a strong effect in at least 1 growth condition (Fig. 4A).

Of the identified sRNAs, many were important for growth in all conditions (i.e. repression of the sRNA reduced growth rate), while some (e.g. *ncl0530* and *ncl0320*) had more condition-dependent effects (Fig. 4A). Interpretation of the fitness scores of these sRNAs is complicated by possible polarity effects, as repression of an sRNA may also affect expression of the surrounding genes. We therefore compared the fitness score of each sRNA with the fitness score of the colocalized genes (upstream and downstream) in each condition (Fig. 4, B and C). This comparison revealed only 2 ncRNAs that had fitness scores independent of those of their surrounding genes, *ncr0050* and *ncr1080* (Fig. 4, D and E). These 2 sRNAs may be in trans to their target and are important for growth in all tested conditions. Ncr1080 (also named SyR47) is located upstream of the lipoprotein nlpD (*slr0993*) but forms an independent transcriptional unit that is upregulated in high light (Kopf et al. 2014). Ncr0050 is not previously characterized.

### Prediction of sgRNA efficacy from sequence

Most genes in the *Synechocystis* CRISPRi library were targeted by 5 sgRNAs. This redundancy made it possible to correlate fitness scores (a measure of sgRNA efficacy) of each sgRNA with its nucleotide sequence. It was shown previously that the efficacy of the guide RNA in mediating DNA cleavage by the Cas9 nuclease varies and that some of this variation can be attributed to the binding properties of the RNA/DNA hybrid (Xu et al. 2015; Labuhn et al. 2018; Peng et al. 2018; Xiang et al. 2021). Consequently, numerous algorithms have been derived in these and similar studies in order to predict the efficacy of guide RNAs (Liu et al. 2020), most recently using deep learning approaches (Wang and Zhang 2019; Xiang et al. 2021). Most of these studies focused on the CRISPR/Cas9 DNA cleavage system and eukaryotic hosts, while prokaryotes and CRISPRi repression systems each have their own sequence requirements (Xu et al. 2015; Wang and Zhang 2019).

**Figure 5.**
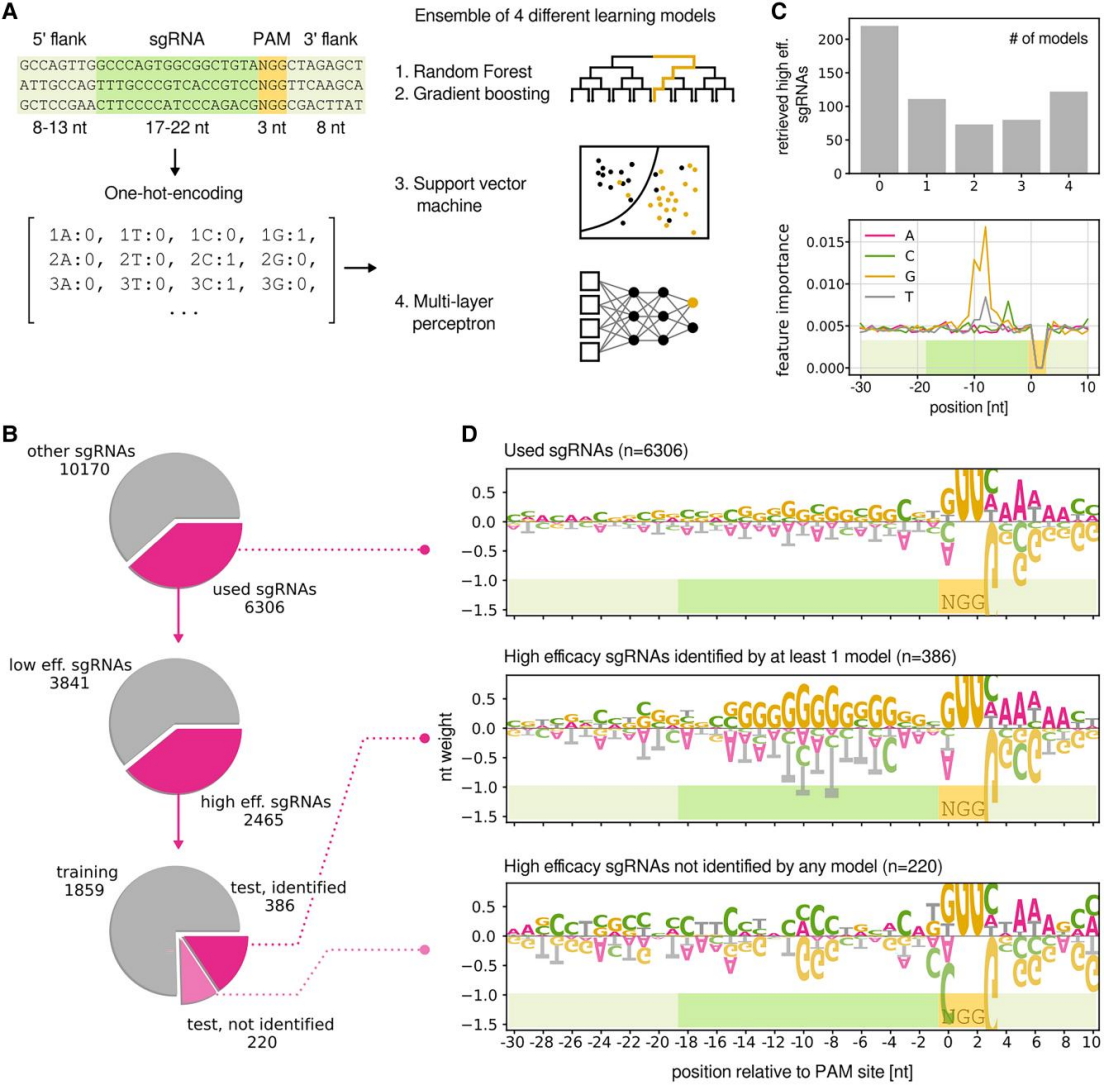
Prediction of sgRNA efficacy from sequence. **A)** Schematic overview of model training and prediction. sgRNA sequences were “1-hot-encoded” (converted to binary vectors of 0 and 1), split into training and validation sets, and used to train 4 different learning models. **B)** Subsets of sgRNAs used for model training and testing. Of the 6,306 sgRNAs included in modeling, 3,841 were labeled as high efficacy and 2,465 as low efficacy. Of the 606 high-efficacy sgRNAs in the validation set, 386 were labeled correctly by at least 1 model. **C)** Number of correctly labeled sgRNAs in the validation set by number of models (upper panel). Feature importance in nucleotide species per sequence position derived from random forest (lower panel). See Supplemental Fig. S11 for the equivalent feature importance derived from gradient boosting. **D)** Sequence logos visualizing the weight of each nucleotide at each position (Tareen and Kinney 2020). Height of letters corresponds to the probability of encountering the respective nucleotide. Positive values, enrichment; negative values, depletion.

Here, we leveraged the information obtained in our extensive fitness screening to predict sequence motifs that lead to better or worse repression. To this end, sgRNA sequences including 8 to 12 nt of the 5′ and 3′ genomic context were selected as features for statistical learning, together with 5 additional features derived from the sgRNA as a whole (GC content, melting temperature, length, distance to promoter, and the “crisproff” score which estimates the binding energy of the DNA-sgRNA hybrid) (Alkan et al. 2018). Features were used to train an ensemble of 4 different models (random forest, gradient boosting machine, support vector machine, and multilayer perceptron; Fig. 5A). The models were trained to predict a binary classifier based on the repression efficacy *E* (low, 0 ≤ *E* ≤ 0.5; high, 0.5 < *E* ≤ 1).

From the complete set of gene-targeting sgRNAs (*n* = 16,476), we included only those where the targeted gene had any effect on fitness in at least 1 condition, even if the effect was small (abs(fitness) > 1, *n* = 6,30; Fig. 5B). This comparatively low fitness cutoff was chosen to include a sufficient number of sgRNAs for training. Of these sgRNAs, 3,841 were labeled as low-efficacy and 2,465 as high-efficacy sgRNAs. This data set was further split into a training and a validation set containing 75% and 25% of the data, respectively. Models were built and trained using the Python packages scikit-learn and keras/tensorflow (Materials and methods), and model quality was evaluated by comparing the predicted sgRNA class with the actual class in the validation set. In terms of sensitivity (ability to retrieve high-efficacy sgRNAs), the best performing model was the support vector machine followed by the multilayer perceptron, while decision tree–based models had problems retrieving high-efficacy sgRNAs (Supplemental Fig. S10A and Table S1). Of the 606 high-efficacy sgRNAs in the validation set, 386 were correctly labeled by at least 1 out of 4 models (Fig. 5, B and C), while 220 were not correctly labeled by any model. Overall model performance was nevertheless far from perfect: on average, only 41% of the high-efficacy sgRNAs were correctly identified. Several problems might contribute to difficulties in identifying a certain subset of high-efficacy sgRNAs: the type or number of features is too limited, the number of observations (sgRNAs) is too low for the models in order to pick up important patterns during training, and unknown or random regulatory events in the cell influence sgRNA efficacy.

Next, we were wondering which features were most important for the models to classify sgRNAs. The 2 decision tree–based models (random forest and gradient boosting machine) record feature importance, here meaning the influence of every base at every position in the sgRNA as well as its direct neighborhood (Figs. 5C and S11). Positions −4 to −12 were of particular importance, more specifically a cytosine residue (C) at position −4, a stretch of guanine residues (G) from positions −5 to −12, and a stretch of thymidine residues (T) from positions −7 to −10. Inspecting the sequence motifs of the different sgRNA classes using logo plots revealed a clear enrichment of sequence patterns (Fig. 5D). While a pool of all used sgRNAs showed only a slight preference for G/C for the entire length of the sequence, high-efficacy sgRNAs that were identified by at least 1 model (*n* = 386) showed a strong enrichment of G and a depletion of T in positions −5 to −11, in accordance with feature importance. Interestingly, high-efficacy sgRNAs that were not identified showed a less specific pattern, explaining the difficulty for models to retrieve them correctly.

From the 5 additional features calculated from the entire sequence, 3 were contributing to sgRNA classification: the distance to the promoter was the most important feature, followed by “crisproff” score and melting temperature (Supplemental Fig. S10B). Interestingly, sgRNA length and GC content had little importance, although the previously mentioned G-stretch was a prominent sequence motif. We next validated that GC content was not an important trait differentiating high- and low-efficacy sgRNAs (Supplemental Fig. S10C). The 2 groups had only a marginal difference in average GC content (55% and 53%, respectively). The difference between correctly identified and unidentified high-efficacy sgRNAs was higher though (57% and 51%, respectively). We conclude that G enrichment and T depletion in the “seed region” (first 12 bases preceding the PAM site) is a good predictor for sgRNA efficacy, although not the only 1.

## Discussion

Cyanobacteria are photosynthetic organisms that play a crucial role in the global carbon cycle and have been intensively used as a proof-of-concept microorganism for sustainable production of valuable chemicals. Cyanobacteria are thought to be the ancestors of chloroplast; thus, understanding cyanobacterial gene essentiality and robustness tradeoff mechanisms in response to environmental changes is specifically useful to guide metabolic engineering of all photosynthetic organisms for various biotechnological applications. Compared with gene knockout libraries, e.g. transposon library, our inducible genome-scale CRISPRi gene repression library allows the assessment of gene essentiality and important, fine-tuned regulatory mechanisms in different conditions.

### Fitness tradeoffs and gene essentiality exposed by the CRISPRi library

By performing growth competition cultivations across 11 conditions, we were able to identify condition-specific essentiality of many genes, including those with unknown function (Supplemental Fig. S1 and Data Set 2). These genes would be a high priority for future study for the cyanobacterial community. Comparison across conditions also revealed unexpected fitness contributions of known genes. Our findings on Gap1/2 are a good example of the predictive power of CRISPRi libraries. The low importance of Gap1 is in contradiction to a previous, widely cited study reporting that a Gap1 mutant completely lost the capacity to metabolize glucose (Koksharova et al. 1998). However, our result is supported by earlier studies which showed that the *Synechocystis* Gap1 is phylogenetically closer related to the plant cytosolic GapC, does not show GAPDH activity in vitro, and is expressed at a low level (Valverde et al. 1997; Jahn et al. 2018). Therefore, cyanobacterial Gap2 may possess both chloroplastic NADPH-dependent GAPDH activity (Figge et al. 2000) and cytosolic NADH-dependent GAPDH function, while the existence of Gap1 may be an evolutionary preparation (Martin and Cerff 2017).

The suboptimal regulations we observed were mainly for proteins within light harvesting and conversion: PBS, PSII subunits, and Flv2/4 proteins. The improved growth upon repression of PBS could be due to reduced electron pressure in the “HC and HL” and photoheterotrophy condition, as even quenched PBS still allows 60% of absorbed energy to reach the reaction centers (Kirilovsky 2015). Antennae knockout was also shown to increase PSII and decrease PSI content in *Synechocystis* (Luimstra et al. 2019). An alternative hypothesis is that repression of PBS provides a benefit on protein economy, as predicted by metabolic modeling (Jahn et al. 2018). ApcAB and CpcAB are the most abundant PBS proteins (Supplemental Fig. S3), so their repression would free the most proteome space for additional ribosomes to fuel growth in the “HC and HL” condition where cells grew fastest. Furthermore, repression of CpcC, a linker protein of low abundance but important for antenna light harvesting (Lea-Smith et al. 2014), did not show a growth increase. However, repression of ApcE, a protein that may anchor Apc discs to the thylakoid and is much smaller than ApcAB (Domínguez-Martín et al. 2022), showed similar, if weaker, fitness profiles as ApcAB. The similarity suggests that a reduced antennae connectivity and not necessarily protein burden may also contribute to the growth rate increase, though it is not clear to what extent ApcAB still forms in the ApcE mutant. The growth advantage of repressing PSII subunits and PBS in the photoheterotrophic condition, and an overall weaker effect in the mixotrophic condition, points to the repression of functional light harvesting and conversion as being beneficial.

The observed growth advantage upon repression of Flv2/4 suggests that they are excessively expressed in the 2 extreme C_i_-limiting conditions we tested. In addition to the hypothesis we mentioned in the Results section, we also suspect that our turbidostat cultivation in air-lift photobioreactors with short path length and low cell density resulted in underestimated light fluctuations due to mitigated mixing (Andersson et al. 2019), thus Flv1/3 take more responsibility and Flv2/4 proteins became a burden during light stress. Suboptimal regulation of antennae and alternative electron flow in cyanobacteria is reminiscent of sustained NPQ in plants, which are typically slow in adapting to stress due to low expression of recovery enzymes or other factors (Malnoë 2018). While photoprotection mechanisms in plants are more diverse than in cyanobacteria, there are some parallels, such as the recently discovered slowly relaxing photoprotective quenching (qH) which may have a homolog in cyanobacteria (Gorbunov et al. 2011; Staleva et al. 2015; Amstutz et al. 2020). The use of a CRISPRi sgRNA library in the background of cyanobacteria strains deficient in known photoprotection mechanisms could identify additional players.

### Limitations of CRISPRi repression libraries

This work improves significantly upon a previous CRISPRi library (Yao et al. 2020), both in terms of growth conditions screened and in library quality. The inclusion of 5 sgRNAs targeting each gene was critical for a confident assessment of gene fitness. The binding efficacy of sgRNAs is highly sequence dependent, and off-target binding is a significant challenge. For example, an analysis of dCas9 binding in *E. coli* found that sgRNAs with PAM regions containing a 9 nt identity elsewhere were likely to bind off-target (Cui et al. 2018). Therefore, the effect of all sgRNA clones for a target gene should be considered when determining fitness scores. We found that the multiple-hypothesis–adjusted *P* value (*P*_adj_) associated with each fitness score was a good filter to prevent false conclusions from single outlier sgRNAs with strong fitness effects. In cases where the fitness score was associated with a low *P*_adj_ (high significance), the fitness effect could be reproduced by gene knockout (e.g. Fig. 3, B to D), but not in cases where *P*_adj_ was high (low significance; Supplemental Fig. S7). Because the gene repression by CRISPRi is on the transcriptional level, the fitness score of neighboring, cotranscribed genes must also be considered when interpreting the fitness score of a gene of interest. For example, the fitness score of the last gene in a 2-gene operon depends solely on the repression of that gene, while the fitness score for the upstream gene represents a combination effect from repressing both genes. Only if these 2 fitness scores are not identical, can we say that the upstream gene has a fitness effect upon repression.

A complication of using CRISPRi libraries is that gene repression is not total, meaning residual protein may be retained so that a phenotype is not observed. A survey of previous studies measuring the repression of transcripts by the CRISPRi expression system in *Synechocystis* shows a median repression of 88% (*n* = 19, Sd 9.7%), as measured by RT-qPCR or RNA-Seq, while 2 examples of measuring protein abundance showed reduction by 80% and 95% (Supplemental Table S2) (Yao et al. 2016; Kaczmarzyk et al. 2018; Shabestary et al. 2018; Yao et al. 2020; Shabestary et al. 2021; Behle et al. 2022). Residual protein remaining after repression can complicate interpretation in some cases. For example, the repression of PsaK2 (*sll0629*) did not show a fitness change in any condition (Supplemental Fig. S2A), even though PsaK2 was identified as an essential element involved in state transition–based NPQ during acclimation to high light (Fujimori et al. 2005). Under high-light conditions, expression of *psaK2* is highly elevated to assist energy transfer from PBS to PSI. This upregulation may result in an excess amount of protein to ensure cell robustness in light stress conditions, and CRISPRi repression mutants may retain a basal level of PsaK2 that is sufficient to avoid a growth penalty.

Another confounding variable with a pooled library is that mutant strains competing against each other can also affect the growth of their neighbors. Two cases of cross-talk can be considered: first, a mutant might gain the ability to secrete a compound that is either toxic, beneficial, or by other means affecting fitness of the population. This effect is negligible as long as all strains are relatively evenly distributed and effector molecule concentration is extremely low. The second case is more relevant: a strain that loses the capability to synthesize a certain metabolite but it is provided by the rest of the population. In this case, the repression mutant has no fitness defect and the essentiality of the gene would remain undiscovered.

Furthermore, another consideration with pooled CRISPRi libraries is strain stability. Individual strains in the library are generally stable over time, as both the dCas9 enzyme coding sequence and the sgRNA are integrated into the genome and controlled by an inducible promoter. Evolutionary pressure to mutate either enzyme or sgRNA is low as long as the expression is not induced. In a previous paper, we could show that, without induction, composition of the library does not change significantly over time (up to 30 generations tested) (Yao et al. 2020). Nevertheless, experiments were always started with fresh aliquots of the deep-frozen initial library culture to prevent “passaging” effects. The library can therefore be safely (re-) cultivated or sent to other labs for reuse.

### Guide RNA design principles for cyanobacteria

The retrospective analysis of sgRNA efficacy using our comprehensive fitness data revealed properties important for sgRNA design in cyanobacteria. Firstly, sgRNA design principles for dCas9-mediated CRISPR interference need to be different from principles used for Cas9-mediated DNA cleavage. Similar to the study from Xu et al. (2015), Labuhn et al. (2018), Peng et al. (2018), and Xiang et al. (2021), we found that a region in the center of the spacer (−4 to −12) is most important for sgRNA efficacy, rather than the 4 most proximal positions to the PAM site which were deemed important for Cas9-mediated cleavage (Xu et al. 2015; Labuhn et al. 2018; Peng et al. 2018; Wang et al. 2019; Xiang et al. 2021). The importance of the central region was reported previously but only in conjunction with high GC content (Labuhn et al. 2018). Here, the region showed a marked preference for G but not C, and in-depth analysis of sequence features confirmed that GC content alone is not an important variable. The genomic context surrounding the spacer played no role in determining guide efficacy.

From the higher-order features, distance to the promoter was most important (Xu et al. 2015; Labuhn et al. 2018; Peng et al. 2018; Xiang et al. 2021), followed by the “crisproff” score (Alkan et al. 2018). The effect of promoter distance was stronger in our library than in a comparable pooled CRISPRi library for *E. coli* (Garst et al. 2017; Price et al. 2018; Wang et al. 2018; Yao et al. 2020; Jahn et al. 2021; Vo et al. 2021). For prediction of sgRNA efficacy from plain sequence, the distance to the promoter might not be known. We recommend to select sgRNAs that target within 100 nt downstream of the start codon, where efficacy is highest. The promoter itself or the 5′ untranslated region (UTR) was not targeted in this study, although other studies successfully targeted these elements.

## Materials and methods

### sgRNA library design

Up to 5 sgRNAs were designed for each ORF and ncRNA. ORFs were retrieved from the National Center for Biotechnology Information (NCBI) (reference genome assembly ASM972v1, accessed on March 18, 2016), and ncRNA locations were obtained from Kopf and Hess (Kopf and Hess 2015) as previously described (Yao et al. 2020). An in-house Python script available at (https://github.com/KiyanShabestary/library_designer) was used to design protospacer sequences using the following criteria: GC content between 40% and 80%, absence of bad seeds (Vigouroux and Bikard 2020), absence of G_6_ and T_4_, and length between 18 and 23 bp. Target sequences were searched with the pattern 5′-[CCN]-(N_18_-N_23_)-3′ on the coding strand (NGG PAM). Off-targets were screened on both forward and reverse strands for sites containing either the canonical NGG or the alternative NAG PAMs. Guiding RNAs with homologies at most 1 mutation away in the 15 bp adjacent to the PAM were discarded. For a given ORF or ncRNA, guiding RNAs that were at least 5 bp from 1 another were considered.

### Genetic construction of sgRNA library

The library was inserted in a *Synechocystis* sp. PCC 6803 base strain containing a tetR_PL22_dCas9_SpR expression cassette genome integrated at the psbA1 locus. The sgRNA oligos were synthesized on 2 12 K chips (Custom Array Inc., USA), pooled together in equimolar ratio. The sgRNA oligos were then cloned into a modified pBR322 vector targeting the *slr0397* locus using Golden Gate assembly, as previously described (Yao et al. 2020). The ligation mix was transformed into NEB 10-beta competent *E. coli* cells, and ∼1,000,000 colonies were obtained. Colonies were collected in LB, pooled, and grown overnight. Plasmid DNA was extracted using the ThermoFisher Maxi plasmid extraction kit. Plasmid (10 *µ*g) was transformed in *Synechocystis* tetR_PL22_dCas9/SpR base strain via natural transformation. After cultivating for 10 days at 30 °C and constant illumination of 100 *µ*mol photons m^−2^ s^−1^ from Osram Fluora T8 L 36W/77 light tubes, colonies were collected in BG-11 and pooled. The pooled library was stored in 7% *v*/*v* DMSO at −80 °C.

### Turbidostat cultivation in photobioreactors

The *Synechocystis* sgRNA library was cultivated in 8-tube Multi-Cultivator MC-1000-OD bioreactors (Photon System Instruments, Drasov, CZ) with 65 mL culture volume per tube. Temperature (30 °C), constant light intensity (from back side of the photobioreactor), and turbidostat pumping system were controlled by an in-house computer program described in Jahn et al. (2018). A gas mixing system GMS150 (Photon System Instruments, Drasov, CZ) was used to provide 1% *v*/*v* CO_2_ for HC conditions and air otherwise. Gas bubbling rate was manually set to an average of 90 bubbles per min, with a total flow rate of 100 mL min^−1^ per reactor tube. Fluctuating light (1,500 *µ*mol photons m^−2^ s^−1^) was provided by an extra LED light panel, PARADIGM LIGHT WH 1200-V (Beambio), from the front side of the photobioreactor. Culture OD_720__nm_ and OD_680 nm_ were automatically measured every 15 min by the photobioreactor, and the turbidity threshold was set to OD_720__nm_ = 0.2. Once the threshold was exceeded for 3 measurements in any tube, 5 mL fresh medium was pumped into the tube for dilution. All cultures were initially cultivated in turbidostat at a standard condition (30 °C, 60 *µ*mol photons m^−2^ s^−1^, BG11 [pH = 7.8] medium with 25 *µ*g mL^−1^ spectinomycin, 25 *µ*g mL^−1^ kanamycin, and 0.5 *µ*g mL^−1^ aTc) for 48 h to allow sufficient repression; after that, specific conditions (see Table 1) were applied. Generation time was calculated as *T*_gen_ = ln 2/growth rate, and *T*_0_ was the condition switching time point. At 4th, 8th, and 10th generations, cells were harvested by centrifuging 12 mL culture at 4 °C, 3,000 × *g* for 10 min. Supernatant was discarded completely and cell pellets were stored at −20 °C.

### Library preparation and NGS

Genomic DNA was extracted from harvested cell pellets using GeneJET Genomic DNA purification Kit (Thermo Fisher Scientific), using the protocol for Gram-positive bacteria due to the dense cyanobacterial cell wall. Extracted gDNA was used as template for the 1st PCR to amplify sgRNA region (with primers: LUYA593 5′-ACACTCTTTCCCTACACGACGCTCTTCCGATCTCAGTGATAGAGATACTGGGAGC-3′ and LUYA594 5′- GACTGGAGTTCAGACGTGTGCTCTTCCGATCTGCCTTATTTTAACTTGCTATTTCTAG-3′) and add NGS adaptors. PCR products were purified using AMPure XP beads (BECKMAN COULTER) and used as template for the 2nd PCR where Illumina barcodes were added by NEBNext Multiplex Oligos for Illumina (Dual Index Primers Set 1 and 2) (New England Biolabs). PCR products were purified using AMPure XP beads (BECKMAN COULTER) and quantified by the Qubit 4 Fluorometer (Thermo Fisher Scientific). Samples were pooled together such that the final concentration was equal (100 ng µL^−1^), and the pooled library was purified from agarose gel using GeneJET Gel Extraction Kit (Thermo Fisher Scientific). Two rounds of NGS were carried out on an Illumina NextSeq 2000 system using NextSeq 2000 P3 kit (50 cycles), with 72 samples sequenced simultaneously per round.

### Fitness score calculation

Sequencing data were preprocessed using an automated pipeline available at https://github.com/MPUSP/nf-core-crispriscreen. Sequencing data were retrieved from Illumina’s base space server using the bs-cp tool. Fastq.gz files were quality trimmed using Trimgalore v0.6.7 (https://www.bioinformatics.babraham.ac.uk/projects/trim_galore/). Read and run quality was summarized using FastQC v0.11.9 (https://www.bioinformatics.babraham.ac.uk/projects/fastqc/) and MultiQC v1.12 (https://multiqc.info/). Reads from filtered fastq.gz files were aligned to the reference genome (fasta file with all sgRNA sequences) using Bowtie2 v2.4.4 (Langmead et al. 2019) with option -U (unpaired reads). The output from Bowtie2, sequence alignment files (SAM files), were further processed by samtools (Danecek et al. 2021) using view and sort commands. Counts of mapped reads per sgRNA were calculated using subread/featureCounts v2.0.1 (https://nf-co.re/modules/subread_featurecounts). Next, count tables per sample were summarized to a single table using a custom R script, and statistics for pairwise sample comparison was calculated using DESeq2 (Love et al. 2014). Log_2_ fold change values were normalized between conditions using the function normalizeBetweenArrays (method = “quantile”) from the R package limma to account for possible differences in number of generations. Fitness scores for each sgRNA and condition were calculated by determining the area under the curve of the log_2_ fold change (log_2_ FC) over time *t* normalized by total cultivation time.


$$F=\frac{\mathrm{AUC}(t,\mathrm{lo}g_{2}\mathrm{FC})\times 2}{\max(t)}.$$

In the second step, a single fitness score for each gene was calculated from all sgRNAs of that gene by determining the weighted mean, where weight *w_i_* for each sgRNA *i* was based on the correlation coefficient *R_i_* of 1 sgRNA with the others and its repression efficacy *E_i_*:


$$F_{\mathrm{wmean}}=\frac{\sum_{i=1}^{n}\left(F_{i}\times w_{i}\right)}{n};w_{i}=R_{i}\times E_{i}.$$

The repression efficacy *E* is the fitness score of a single sgRNA divided by the maximum fitness of all sgRNAs for the same gene (0 ≤ *E* ≤ 1).

### Statistical analysis

All cultivation and sequencing experiments were performed with 3 biological replicates. Replication was carried out at the stage of bioreactor cultivation (inocula were obtained from single precultures grown in 200 mL shake flasks). *P* values were calculated from a comparison of sgRNA fitness score (*n* = 1 to 5 depending on gene) with nontargeting control sgRNAs (*n* = 10) using the Wilcoxon rank sum test. *P* values were multiple-hypothesis corrected using the Benjamini–Hochberg procedure. For selected tasks, a combined score *S* was calculated for each target and condition by combining effect size (fitness score *F*) and adjusted *P* value according to the formula:


$$S=\mathrm{abs}(F_{\mathrm{wmean}})\times -\mathrm{lo}g_{10}p_{\mathrm{adjust}}.$$

A combined score threshold of 4 corresponds to an absolute fitness score of 2 and an adjusted *P* value of 0.01 and was considered as significant. All analyses of fitness data were performed using the R programming language and are documented in an R notebook available at https://github.com/m-jahn/R-notebook-crispri-lib. Statistical data are provided in Supplemental Data Set 3.

### Construction and cultivation of single knockout mutants

To generate ΔGap1 (*slr0084*), ΔGap2 (*sll1342*), ΔCP12 (*ssl3364*), ΔFlv2 (*sll0219*), and Δ*slr1505* mutants, a chloramphenicol acetyltransferase gene cassette was integrated into corresponding gene locus. Integrating plasmids were designed to have 1,000 bp homologous regions on both upstream and downstream of the target gene. Genotypes of the knockout strains were confirmed using 2 pairs of primers: 1 pair anneals to chloramphenicol cassette to screen colonies, and another pair anneals to the original gene to check segregation. The knockout strains, ΔGap1, ΔGap2, ΔCP12, and Δ*slr1505*, were cultivated in batch mode with initial OD_730_ = 0.1 in multicultivators. The specific conditions used for the batch cultivation were kept identical to the corresponding conditions in turbidostat cultivation.

### Chlorophyll fluorescence measurements

Chlorophyll fluorescence was measured using a pulse amplitude modulated fluorometer Dual-PAM 100. Three milliliters of cell suspensions (in BG11 media pH = 7.8) with final chlorophyll content of 5 *μ*g Chl mL^−1^ were placed in a flat bottom stirred cuvette at darkness for 15 min before measurements. Detection was done at 30 °C using red (618 *µ*mol photons m^−2^ s^−1^) and blue (18 *µ*mol photons m^−2^ s^−1^) actinic light. Fm, maximum fluorescence level, was measured in high red actinic light illumination with 10 *μ*m DCMU addition; F_0_, intrinsic fluorescence level while the cells were exposed to the modulated measuring beam, was measured in low blue light illumination. Fv/Fm = (Fm−Fo)/Fm.

### Oxygen production and uptake rate measurement

Oxygen production and uptake was determined using membrane inlet mass spectrometry (MIMS). Cells grown at air level CO_2_ were collected, centrifuged at 3,000 × *g* for 4 min, and resuspended in fresh BG11 (pH = 7.8) at 10 *μ*g Chl mL^−1^. The cell suspension was then placed into MIMS, and O^18^ was added to monitor oxygen uptake. Red actinic light intensities used are 0, 117, 207, 434, 618, and 989 *μ*mol photons m^−2^ s^−1^. In order to increase data reproducibility, 1.5 mm NaHCO_3_ was added into the cell suspension.

### Machine learning to predict sgRNA efficacy

The machine learning pipeline was implemented as a Jupyter Notebook available at https://github.com/m-jahn/R-notebook-crispri-lib, using Python v3.9.7. In order to predict sgRNA efficacy, sgRNA sequences were imported from a fasta file and mapped back to the genome to retrieve the PAM site and 5′ and 3′ genomic flanks. Flanking sequences were variable in length to allow a fixed total length of 40 nt. Nucleotide sequence was 1-hot-encoded by converting an A, T, C, or G at a single position into a 4-digit binary vector of 0 or 1. Altogether, this resulted in a vector of 160 features for each sgRNA. Only a subset of 6,306 sgRNAs was used for modeling, where the targeted gene showed a fitness score of abs(F) ≥ 1 in at least 1 condition. The target variable for training was the sgRNA efficacy E, ranging between 0 and 1, and experimentally determined from CRISPRi library cultivations. To simplify modeling, E was binned into 2 categories, “low” (E < 0.5) and “high” (E ≥ 0.5). An ensemble of 4 models for classification problems were selected, RandomForestClassifier, GradientBoostingClassifier, and support vector machine (svm) from scikit-learn, as well as a multilayer perceptron (small, fully connected neural network) from keras/tensorflow. The feature array and the target variable were split into training and validation data sets (75% or 4,730 observations and 25% or 1,576 observations, respectively). The first 3 models were tuned using a grid search with the following parameters. Random forest: “n_estimators”: (20, 50, 100, 200, 300, 400), “max_features”: (“auto”, “sqrt”), and “max_depth”: (5, 10, 25, 50, 100). Gradient boosting: “loss”: (“deviance”, “exponential”), “n_estimators”: (100, 200, 300), “learning_rate”: (0.01, 0.05, 0.1, 0.5), and “max_depth”: (1). Support vector machine: “kernel”: (“rbf”, “linear”), “C”: (0.5, 0.75, 1, 1.5, 2), and “gamma”: (0.01, 0.05, 0.1, 0.5, 1, 5). Model training was then performed with the best parameter set. For the multilayer perceptron, different configurations of 1 to 3 hidden layers with 8 to 128 nodes were tested manually, and the best performing configuration was selected. The final topology of the model was 1 input layer with 64 nodes and activation function “relu”, 1 hidden layer with 128 nodes and activation function “relu”, and 1 output layer with 2 nodes and activation function “softmax”. The model was trained with maximally 200 epochs but already converged after <100. The loss function that evaluates model performance during training was “categorical_crossentropy”, and the optimizer was gradient descent with momentum (“tf.keras.optimizers.experimental.SGD”) with learning rate = 0.01 and momentum = 0.9.

### Accession numbers

Raw sequencing data were deposited at the European Nucleotide Archive (ENA accession number ERP144974).

## Supplementary Material

koad208_Supplementary_Data

## Supplemental data

The following materials are available in the online version of this article.

**Supplemental Figure S1**. Bubble plot showing t-SNE clustering of 187 genes which were selected based on absolute fitness score ≥ 4 and adjusted *P* value ≤ 0.01 (Wilcoxon rank sum test).

**Supplemental Figure S2**. Heat map showing fitness score for repression of photosystem genes in *Synechocystis* PCC 6803.

**Supplemental Figure S3**. Percent protein mass of the total proteome for the light harvesting complex (PBS) from *Synechocystis* PCC 6803.

**Supplemental Figure S4**. Photosynthetic parameters of wild-type (WT) *Synechocystis* and *flv2* knockout mutant.

**Supplemental Figure S5**. Fitness score for repression of genes involved in carbon transport.

**Supplemental Figure S6**. Metabolic flux for mixotrophic and photoheterotrophic growth of *Synechocystis* sp. PCC 6803.

**Supplemental Figure S7**. Validation example of a gene with high absolute fitness score but high (insignificant) *P* value.

**Supplemental Figure S8**. Validation of CRISPRi library results for *gap1*, *gap2*, and *cp12* using deletion mutants.

**Supplemental Figure S9**. ncRNAs targeted by the CRISPRi library.

**Supplemental Figure S10**. Additional statistics for sgRNA efficacy machine learning.

**Supplemental Figure S11**. Feature importance in nucleotide species per sequence position derived from gradient boosting model.

**Supplemental Table S1**. Performance metrics of machine learning models.

**Supplemental Table S2**. Examples of repression factors using PL22-dCas9 + PL22-sgRNA in *Synechocystis*.

**Supplemental Table S3**. Reaction names used for metabolic flux mapping in central carbon metabolism of *Synechocystis* sp. PCC6803.

**Supplemental Data Set 1**. Lists of sequences of all the sgRNA used in this study and fitness score of all the sgRNAs and overall fitness scores of all the targeted genes/ncRNAs in all tested growth conditions.

**Supplemental Data Set 2**. Subset of genes where a significant fitness score was obtained in any 1 condition and lists of significant genes for each individual growth condition.

**Supplemental Data Set 3**. Details for the statistics provided in Fig. 3, B to D, and Fig. 4, B and C.
