# Supplementary material for: CRISPR interference screens reveal growth–robustness tradeoffs in Synechocystis sp. PCC 6803 across growth conditions
Source: Plant Cell. 2023 Jul 26;35(11):3937–56. doi: 10.1093/plcell/koad208 (PMC10615215; doi:10.1093/plcell/koad208)
Supplement: koad208_Supplementary_Data [file koad208_supplementary_data.zip › tpc.23.00192Supplemental Figures and Tables.pdf]

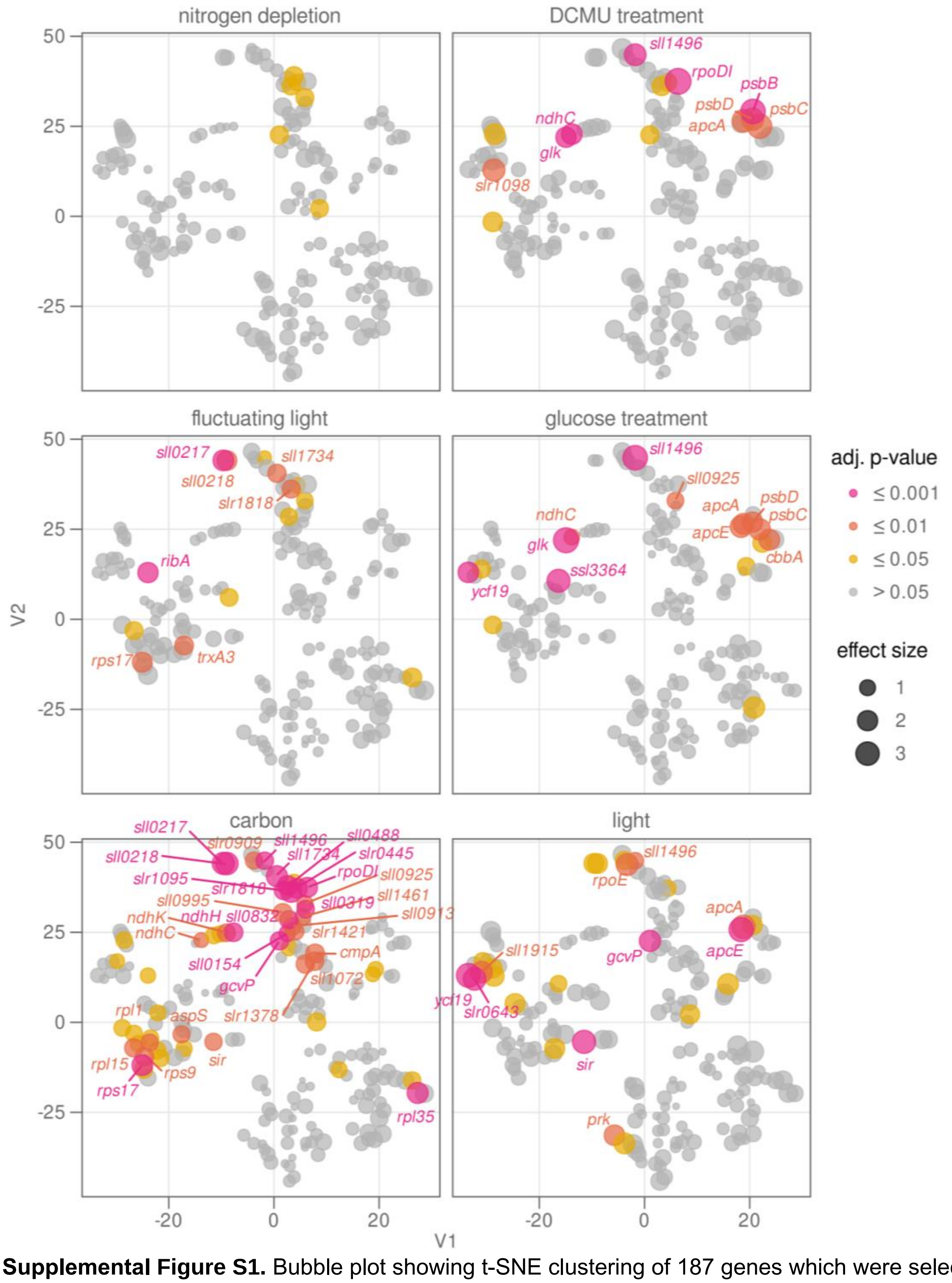

Supplemental Figure S1. Bubble plot showing t-SNE clustering of 187 genes which were selected based on absolute fitness score ≥ 4 and adjusted p-value ≤ 0.01 (Wilcoxon rank sum test). Bubble size encodes effect size of the respective treatment (carbon, light, additional treatments) from multiple linear regression models. Bubble color encodes adjusted p-value from Wilcoxon rank sum test. V1, V2, two dimensions showing gene similarity. This Supplemental Figure supports Figure 1 and identifies potential candidate genes for further analysis.

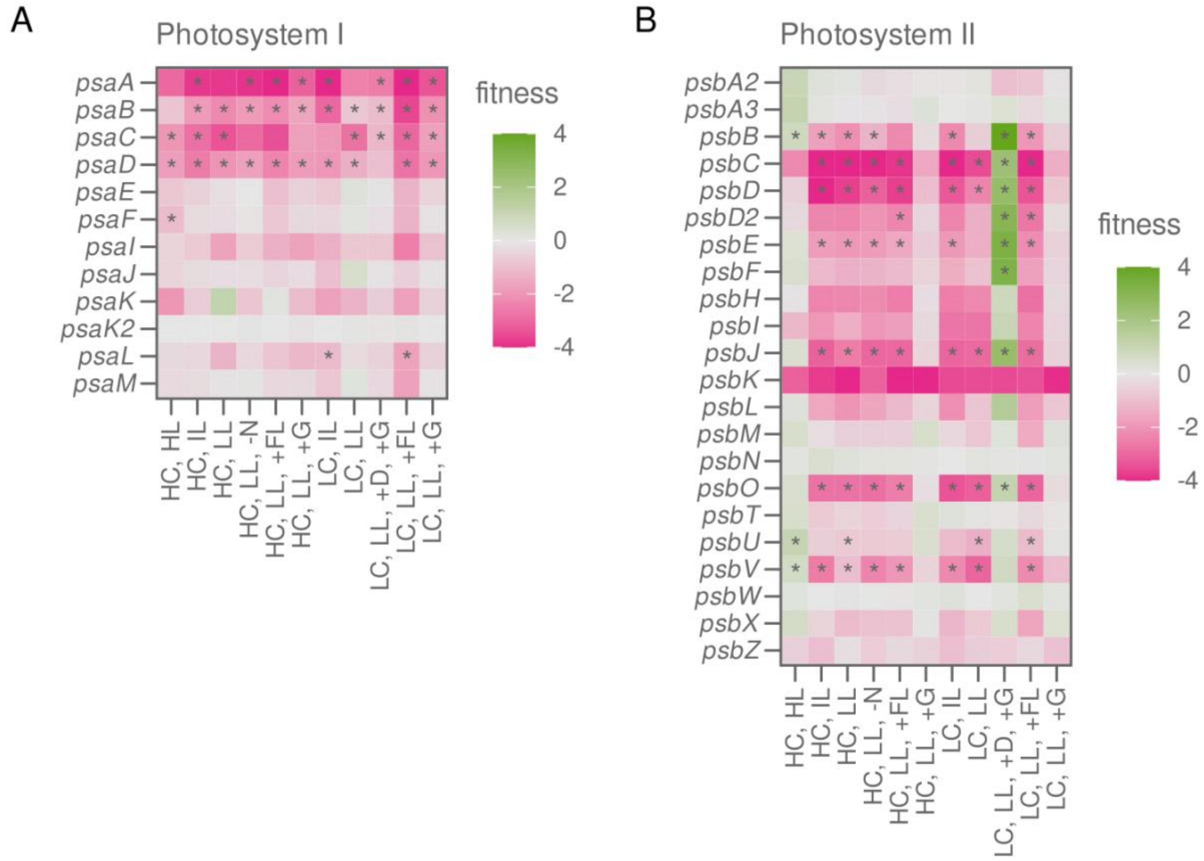

Supplemental Figure S2. Heatmap showing fitness score for repression of photosystem genes in *Synechocystis* PCC 6803. **A)** Genes encoding photosystem I subunits. **B)** Genes encoding photosystem II subunits. Asterisk: Wilcoxon rank sum test, adjusted p-value ≤ 0.01. This Supplemental Figure supports Figure 2 with fitness scores for photosystem genes, another example for the importance of photosynthesis in *Synechocystis* sp. For full names of conditions see Table 1 in the main text.

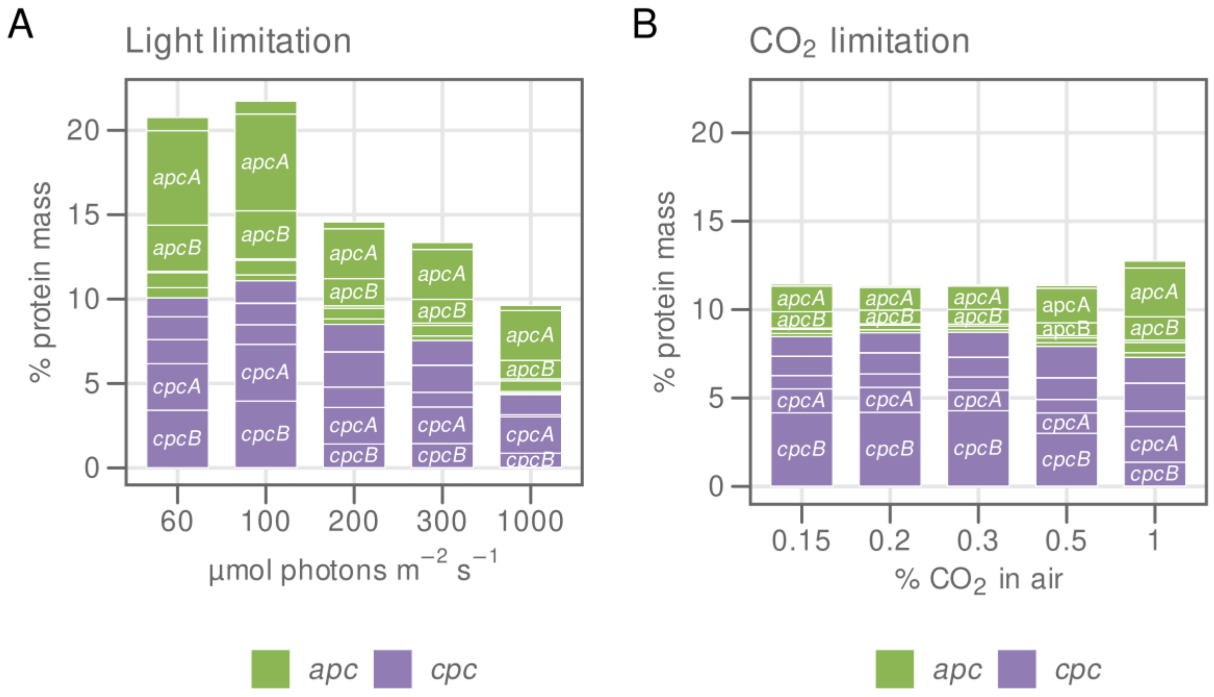

Supplemental Figure S3. Percent protein mass of the total proteome for the light harvesting complex (phycobilisomes) from Synechocystis PCC 6803. Protein mass fraction was estimated from label-free quantification of mass spectrometry data (Jahn et al. 2018). Green, allophycocyanin subunits. Purple, phycocyanin subunits. **A)** Protein mass depending on light limitation. **B)** Protein mass depending on carbon limitation. This Supplemental Figure supports Figure 2 by showing the estimated protein fraction of the largest protein complex by mass, the phycobilisome.

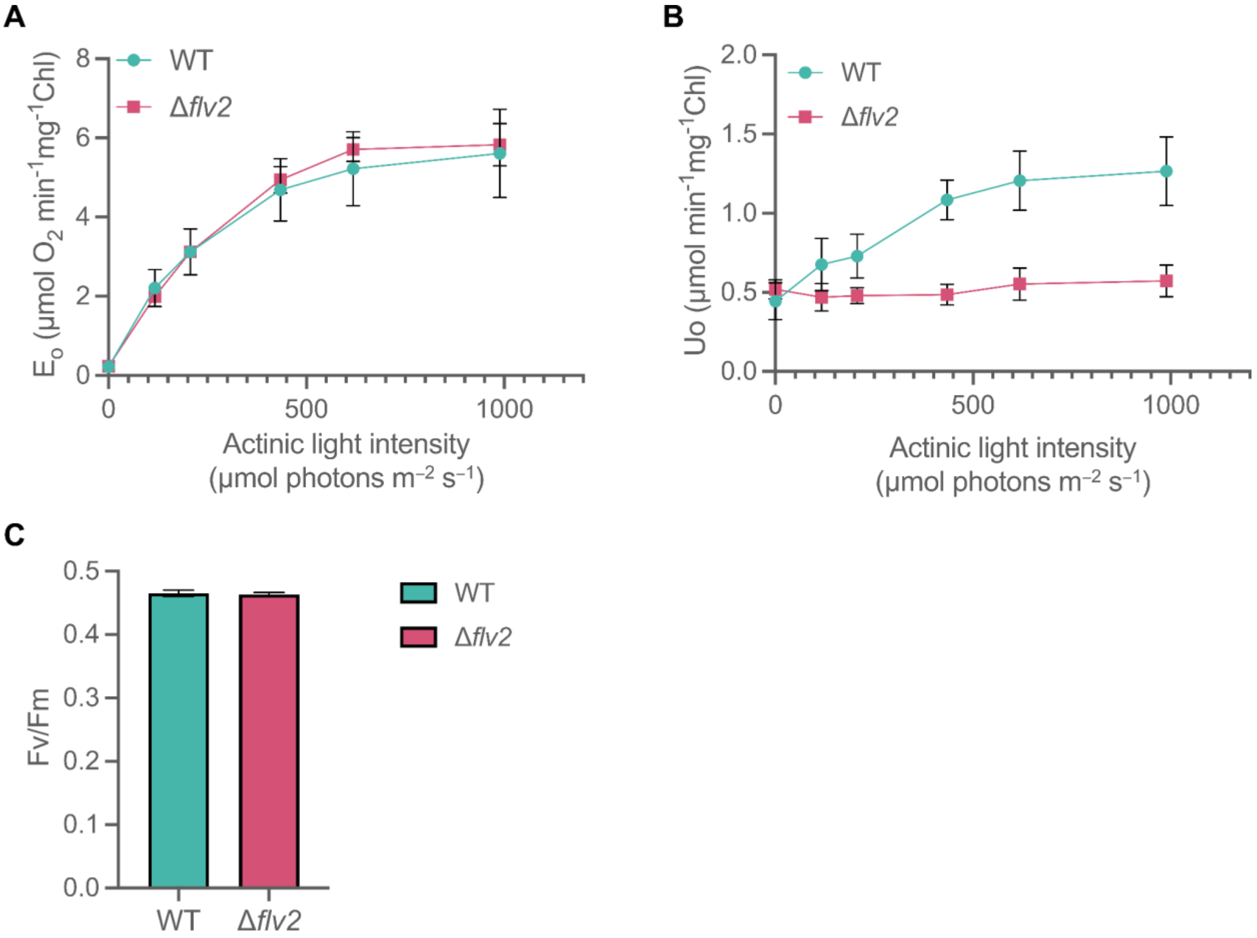

Supplemental Figure S4. Photosynthetic parameters of wild type (WT) *Synechocystis* and *flv2* knockout mutant. Cells were grown in air with 120 μmol photons m^{-2} s^{-1} light for 72 hours before measurements were taken. **A)** Oxygen production rate, and **B)** oxygen uptake rate of WT and Δ*flv2 strains*. Cells were concentrated to a chlorophyll concentration 10 μg mL^{-1} and incubated in darkness for 15 min before each measurement. Red actinic light intensities used were 0, 117, 207, 434, 618, 989 μmol photons m^{-2} s^{-1}. In order to increase data reproducibility, 1.5 mM $NaHCO_3$ was added, and O^{18} was used for monitoring oxygen uptake. **C)** Photosynthetic parameter Fv/Fm of WT and Δ*flv2*. Cells were concentrated to a chlorophyll concentration 5 μg ml^{-1} and incubated in darkness for 15 min before chlorophyll fluorescence measurements on a Dual-PAM-100 spectrometer without addition of $NaHCO_3$. Fv/Fm, represents maximum quantum efficiency of photosystem II. Fm, maximum fluorescence level was measured in high red actinic light illumination with 10 μM DCMU ; Fo, intrinsic fluorescence level while the cells were exposed to low blue light illumination. Two-way ANOVA was conducted, p-value=0.3483. Error bars represent the standard deviation of three biological replicates. This Supplemental Figure supports Figure 2 C.

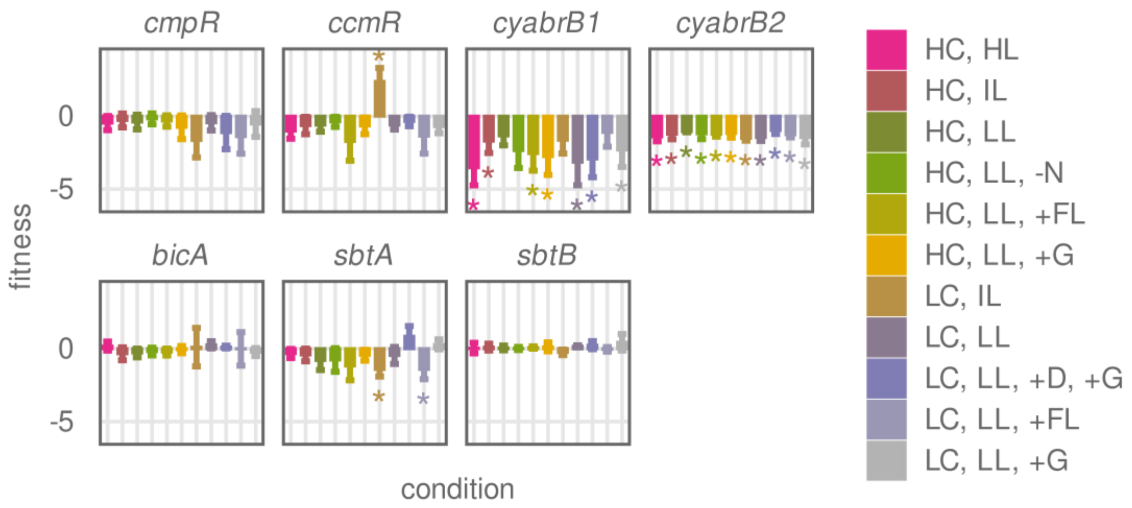

Supplemental Figure S5. Fitness score for repression of genes involved in carbon transport. *CmpR*, *ccmR*, *cyabrB1*, and *cyabrB2* are regulatory genes. *BicA*, *sbtA* and *sbtB* are carbon transporters. Asterisk: Wilcoxon rank sum

test p-value ≤ 0.01. For full names of conditions see Table 1 in the main text. This Supplemental Figure supports Figure 2 D with all remaining genes important for CO2 uptake (regulation).

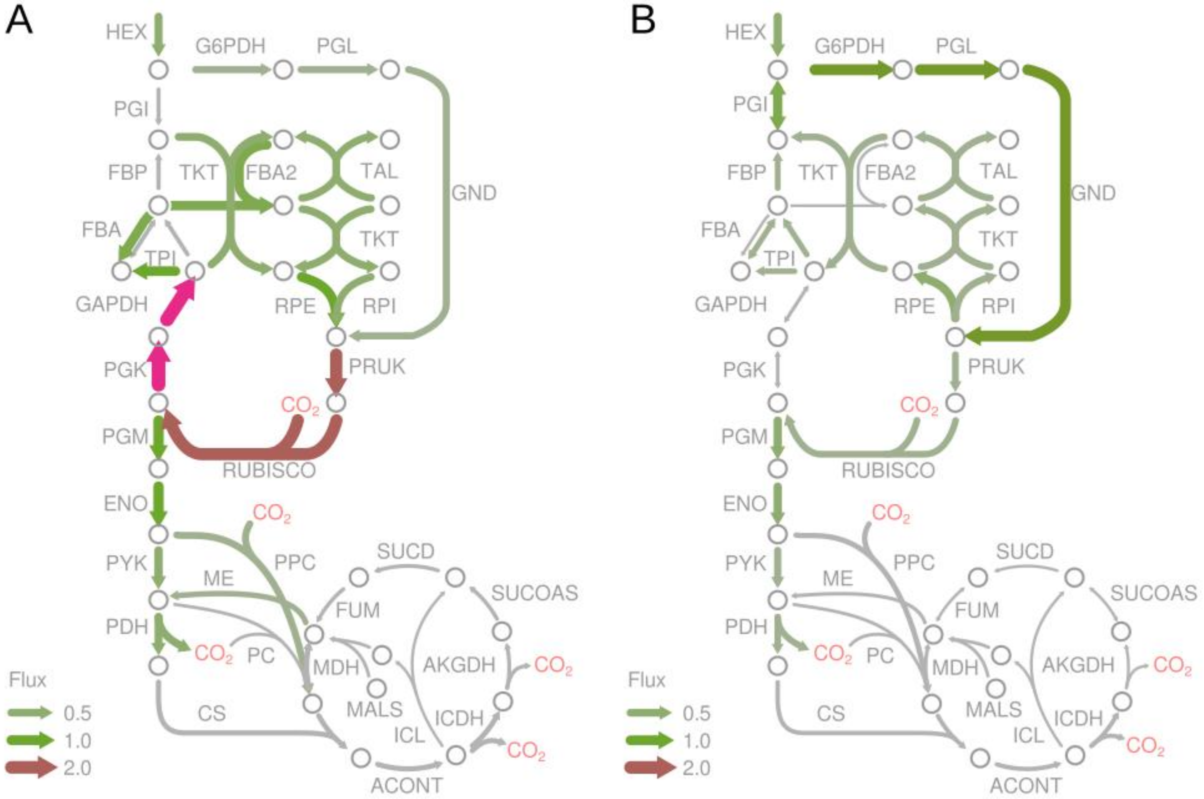

Supplemental Figure S6. Metabolic flux for mixotrophic and photoheterotrophic growth of *Synechocystis* sp. PCC 6803. Fluxes were determined by (Nakajima et al. 2014) using ^{13}C labeled D-glucose. Fluxes were plotted on a custom made metabolic map using the R package fluctuator (*https://github.com/m-jahn/fluctuator*). Reactions and their directionality are shown with arrows and named with capital letters according to the BiGG standard. For full reaction names see Table S3. Arrow thickness indicates the reaction flux in mmol g DCW^{-1} h^{-1}. **A)** Mixotrophy (growth in presence of glucose). **B)** Photoheterotrophy (growth in presence of glucose and DCMU to block photosystem activity). This Supplemental Figure supports Figure 3 A with metabolic flux in two important growth conditions corresponding to the same conditions used for library cultivation in Figure 3 A.

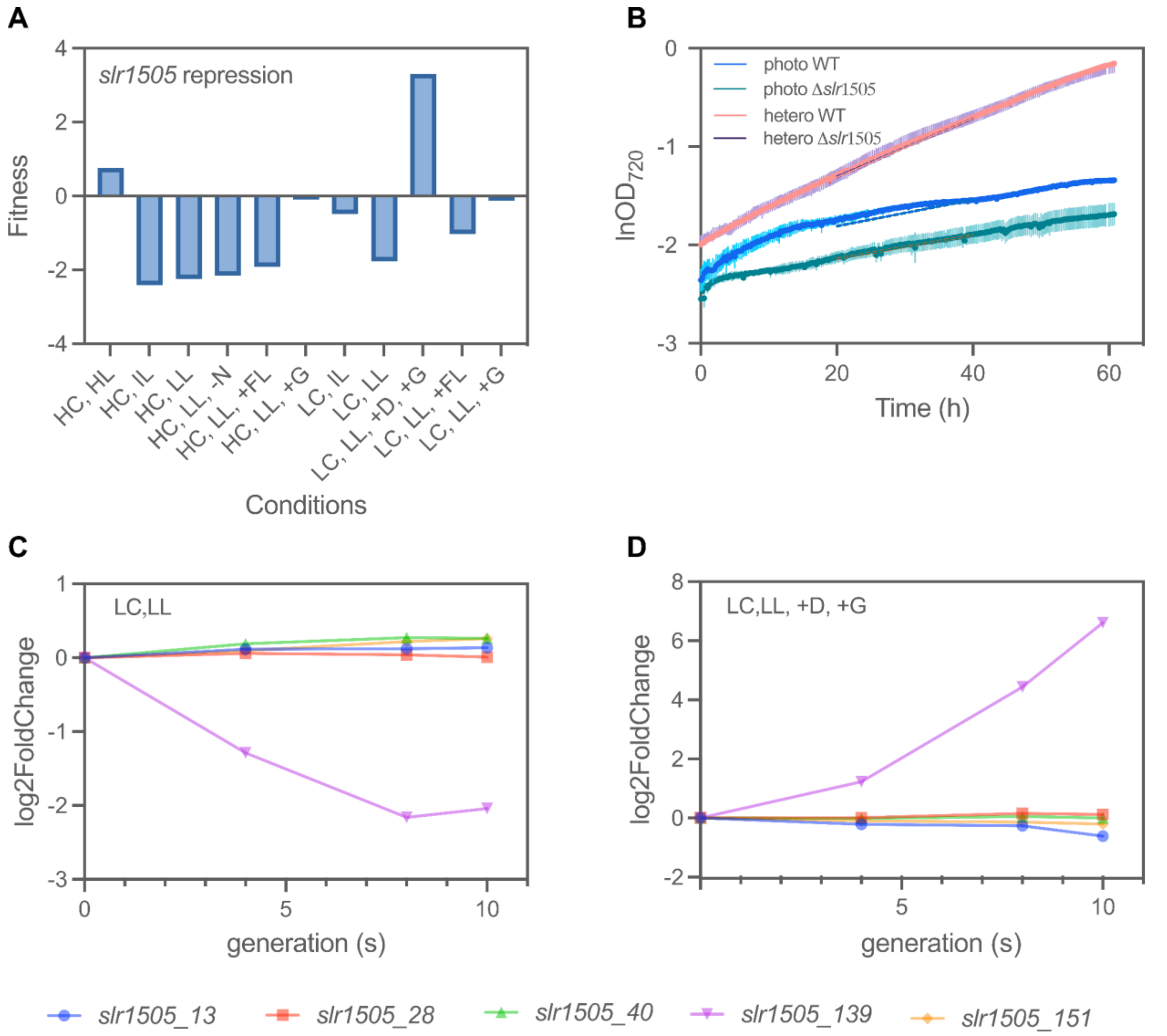

Supplemental Figure S7. Validation example of a gene with high absolute fitness score but high (insignificant) *p*-value. **A)** Fitness scores of *slr1505* in all 11 conditions tested on CRISPRi library. **B)** Natural logarithm-transformed OD_{720} of WT and Δ*slr1505* in 60 hours batch cultivation. **C)** Log_2 fold change of strain abundance over 10 generations with 5 individual sgRNAs targeting *slr1505* in phototrophic condition. **D)** Log_2 fold change of strain abundance over 10 generations with 5 individual sgRNAs targeting *slr1505* in photoheterotrophic condition. For full names of conditions see Table 1 in the main text. This Supplemental Figure supports Figure 3 B-D with an example of a gene whose fitness phenotype could not be validated.

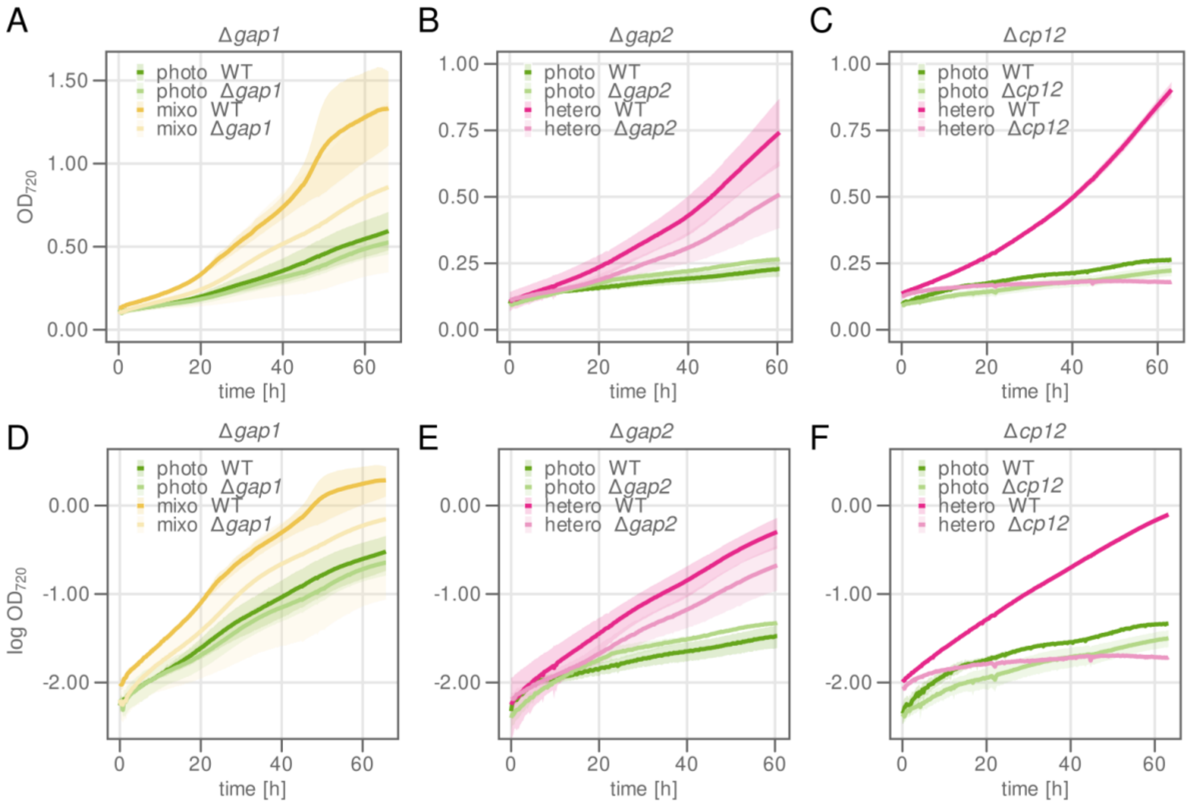

Supplemental Figure S8. Validation of CRISPRi library results for *gap1*, *gap2* and *cp12* using deletion mutants. Cultivation was performed in the same conditions as were used for cultivating the CRISPRi library, but in batch mode instead of turbidostat. WT was used as control in each cultivation. Growth recorded as change in optical density at 720 nm (OD_{720}). Line and ribbons: Mean and standard deviation of at least two biological replicates. **A)** Δ*Gap1* mutant. Phototrophy: HC, LL. Mixotrophy: HC, LL, +G. **B)** As in A) but for Δ*gap2*. Phototrophy: LC, IL. Photoheterotrophy: LC, LL, +G, +D. **C)** As in A) but for Δ*cp12*. Phototrophy: LC, LL. Photoheterotrophy: LC, LL, +G, +D. **D-F)** As in A-C) but natural logarithm-transformed. For full names of conditions see Table 1 in the main text. This Supplemental Figure supports Figure 3 B, C, D, by showing the raw and log-transformed optical density values used to calculate mutant growth rate.

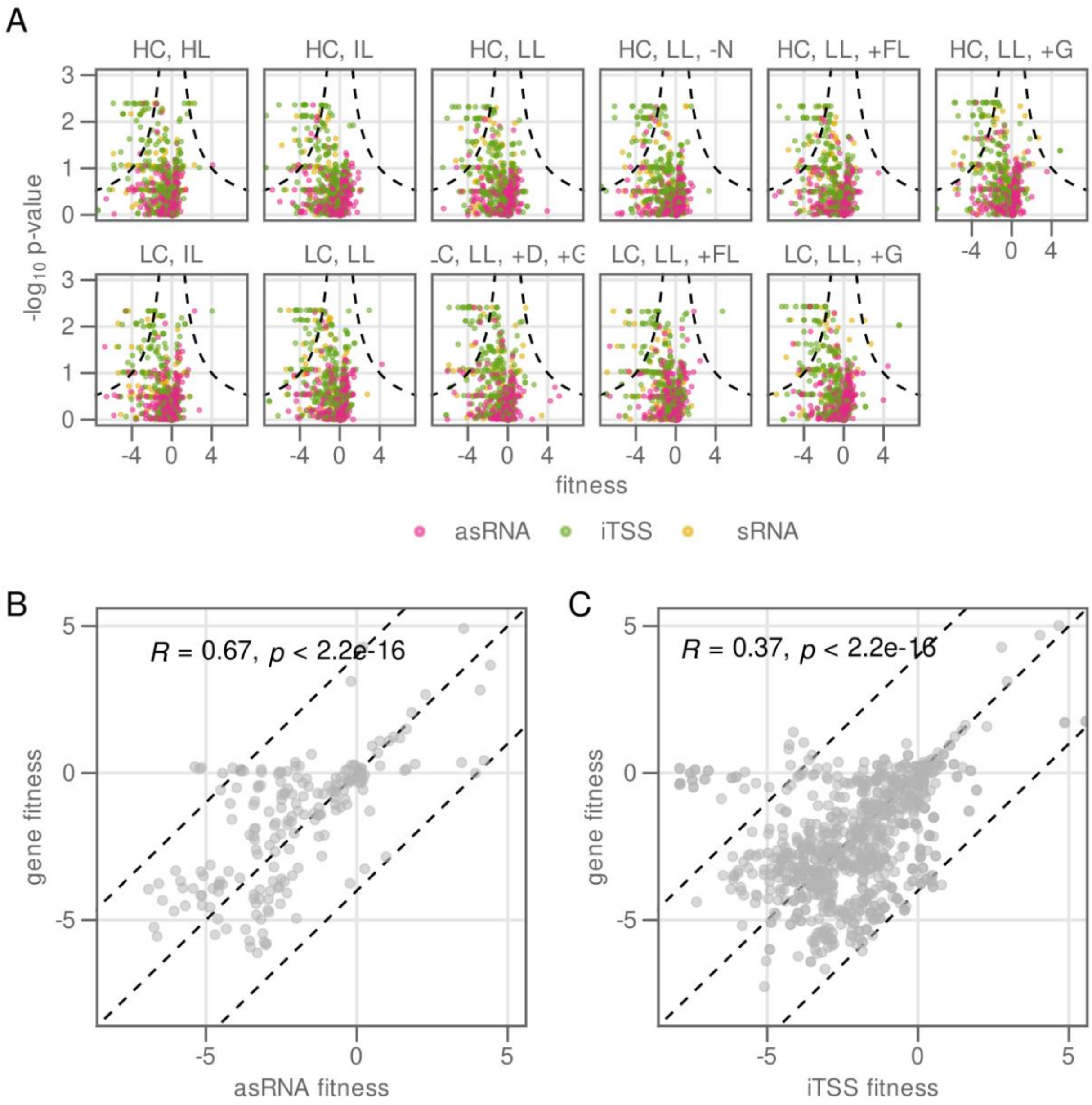

Supplemental Figure S9. Non-coding RNAs (ncRNAs) targeted by the CRISPRi library. **A)** Volcano plot showing fitness (x-axis) and negative $\log_{10}$ p-value from Wilcoxon rank sum test (y-axis) for all ncRNAs, broken down by growth condition and type. asRNA, antisense RNA. iTSS, internal transcription start site. sRNA, small RNA. For full names of conditions see Table 1 in the main text. **B)** Correlation of fitness score of all asRNAs with the fitness score of their respective sense-oriented gene. Every dot represents one asRNA in one growth condition. *R*, correlation coefficient. Dashed lines: boundaries for an absolute fitness difference of 4 between gene and asRNA. **C)** As in B) but for the iTSSs. This Supplemental Figure supports Figure 4 by showing inconclusive fitness results for asRNAs and iTSSs.

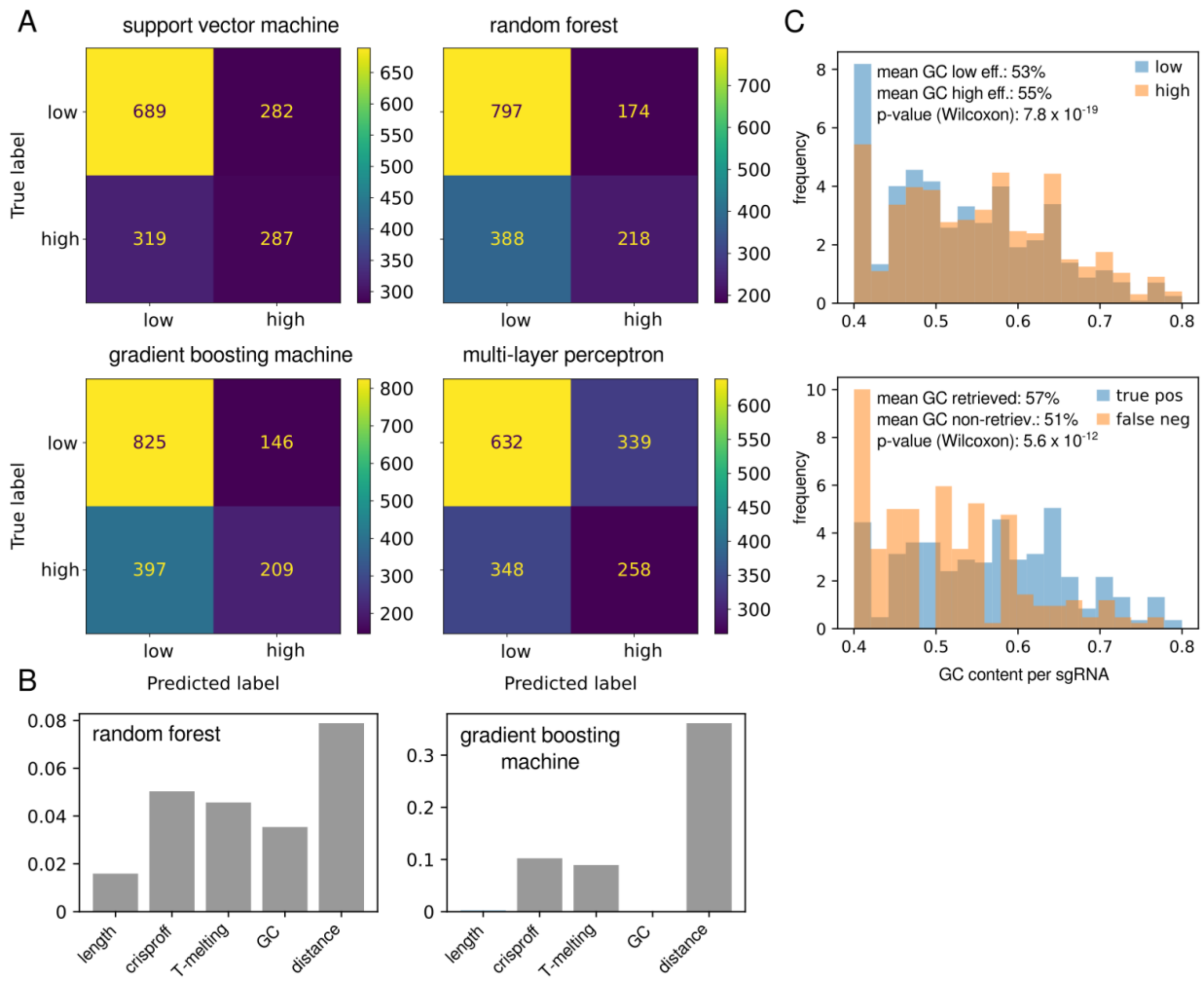

Supplemental Figure S10. Additional statistics for small guide RNA (sgRNA) efficacy machine learning. **A)** Confusion matrices for all four used models showing number of correctly and incorrectly retrieved sgRNA classes. Upper left quadrant: true negatives, upper right: false positives, lower left: false negatives, lower right: true positives. **B)** Feature importance for five additional features derived from sequence or genomic context. length: length of sgRNA (17-22 nt), crisproff: on-target score calculated using the CrisprOff tool at https://github.com/RTH-tools/crisproff (Alkan et al. 2018), T-melting: melting temperature of sgRNA, GC: GC content, distance: distance to the promoter. **C)** Detailed analysis of GC content for different groups of sgRNAs. Upper panel: Histogram of GC content between low efficacy and high efficacy sgRNAs. Lower panel: Histogram of GC content for correctly retrieved (true positive) and incorrectly retrieved (false negative) high efficacy sgRNAs. This Supplemental Figure supports Figure 5 with more detailed metrics from machine learning, including influence of GC content on sgRNA classification.

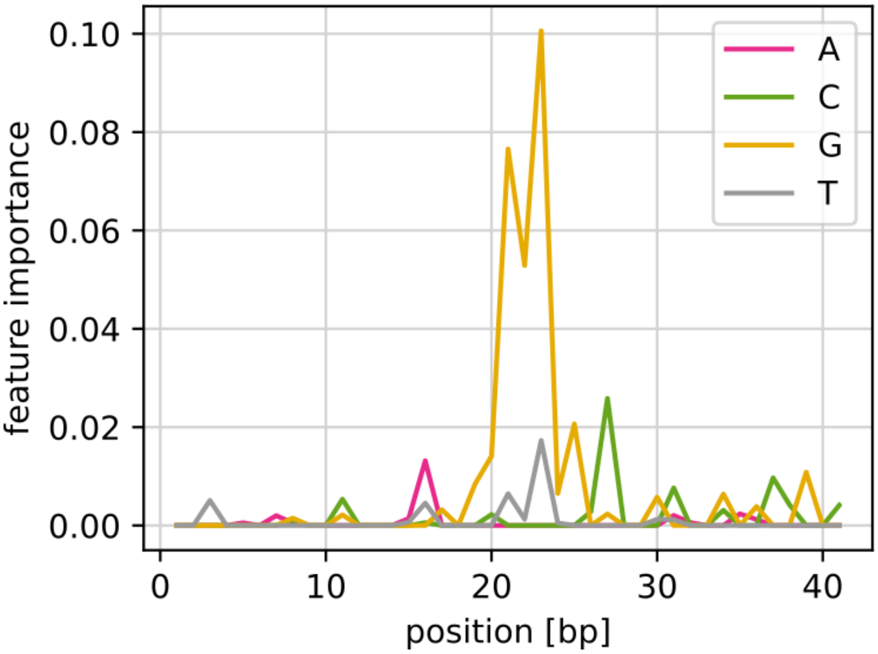

Supplemental Figure S11. Feature importance in nucleotide species per sequence position derived from gradient boosting model. This Supplemental Figure supports Figure 5 C with the equivalent feature importance for the corresponding random forest model.

Supplemental Table S1. Performance metrics of machine learning models. SVM - support vector machine, GBM - gradient boosting machine, RF - random forest, MLP - multi-layer perceptron. Two different classes were predicted, low-efficacy sgRNAs ("0-low") and high-efficacy sgRNAs ("1-high"). * - accuracy is identical for both classes because it is calculated taking both classes into account. This Supplemental Table supports Figure 5 with exact performance metrics for models.

model	class	precision	recall / sensitivity	accuracy*	f1_score
SVM	0-low	0.684	0.710	0.619	0.696
SVM	1-high	0.504	0.474	0.619	0.489
GBM	0-low	0.675	0.850	0.656	0.752
GBM	1-high	0.589	0.345	0.656	0.435
RF	0-low	0.669	0.792	0.630	0.725
RF	1-high	0.527	0.371	0.630	0.436
MLP	0-low	0.651	0.658	0.573	0.655
MLP	1-high	0.443	0.436	0.573	0.439

Supplemental Table S2. Examples of repression factors using PL22-dCas9 + PL22-sgRNA in *Synechocystis*

**Reference**	**Gene target**	**Transcript repression (%)**	**Method**	**Protein repression (%)**
Behle et al., 2022	gyrA	75	RT-qPCR	
Behle et al., 2022	gyrB	75	RT-qPCR	
Yao et al., 2020	*sll1969*	70	RNA-Seq	
Yao et al., 2020	*slr1340*	83	RNA-Seq	
Yao et al., 2020	*slr1916*	97	RNA-Seq	
Yao et al., 2020	*ssl2982*	95	RNA-Seq	
Shabestary et al., 2021	gltA	88	RNA-Seq	80
Kaczymarzyk et al., 2018	plsX	95	RT-qPCR	
Kaczymarzyk et al., 2018	aar	80	RT-qPCR	
Kaczymarzyk et al., 2018	ado	90	RT-qPCR	
Shabestary et al., 2018	pyrF	*75*	RT-qPCR	
Shabestary et al., 2018	odhB	90	RT-qPCR	
Shabestary et al., 2018	gltA	75	RT-qPCR	
Yao et al., 2016	glgC	*75*	RT-qPCR	
Yao et al., 2016	phaEC	90	RT-qPCR	
Yao et al., 2016	*slr0942*	90	RT-qPCR	
Yao et al., 2016	*sll0990*	95	RT-qPCR	

Reference	Gene target	Transcript repression (%)	Method	Protein repression (%)
Yao et al., 2016	*slr1192*	90	RT-qPCR	
Yao et al., 2016	*slr0091*	65	RT-qPCR	
Yao et al., 2016	GFP			94
**Median**		**88**		**87**
**Standard Deviation**		**9.7**		**4.9**

Supplemental Table S3. Reaction names used for metabolic flux mapping in central carbon metabolism of *Synechocystis* sp. PCC6803. This Supplemental Table supports Figure 3 and Figure S6.

**Reaction short name**	**Reaction full name**	**Flux mixotrophy [mmol g DCW-1 h-1]**	**Flux photo-heterotrophy [mmol g DCW^{-1} h^{-1}]**
HEX	Hexokinase (D-glucose:ATP)	0.53	0.58
PGI	Glucose-6-phosphate isomerase	0.03	-0.84
FBP	Fructose-bisphosphatase	0.04	0.16
FBA	Fructose-bisphosphate aldolase	-0.04	-0.16
FBA2	Fructose-bisphosphate aldolase	0.93	0
TPI	Triose-phosphate isomerase	-0.97	-0.16
GAPDH	Glyceraldehyde-3-phosphate dehydrogenase	-2.45	0.01
PGK	Phosphoglycerate kinase	-2.45	0.01
PGM	Phosphoglycerate mutase	1.14	0.6
ENO	Enolase	1.14	0.6
PYK	Pyruvate kinase	0.63	0.39
PDH	Pyruvate dehydrogenase	0.52	0.27
CS	Citrate synthase	0.11	0.05
ACONT	Aconitase	0.11	0.05
ICDH	Isocitrate dehydrogenase	0.11	0.05
SUCD	Succinate dehydrogenase	0.02	0

Reaction	Reaction	Flux mixotrophy [mmol g DCW-1 h-1]	Flux photo-heterotrophy [mmol g DCW^{-1} h^{-1}]
FUM	Fumarase	0.02	0
MDH	Malate dehydrogenase	-0.13	-0.01
G6PDH	Glucose 6-phosphate dehydrogenase	0.37	1.35
PGL	6-phosphogluconolactonase	0.37	1.35
GND	Phosphogluconate dehydrogenase	0.37	1.35
RPE	Ribulose 5-phosphate 3-epimerase	-0.99	0.68
RPI	Ribose-5-phosphate isomerase	0.44	-0.37
TKT1	Transketolase	-0.52	0.33
TKT2	Transketolase	-0.48	0.35
TAL	Transaldolase	0.45	0.35
PRUK	Phosphoribulokinase	1.81	0.3
RUBISCO	Ribulose-bisphosphate carboxylase	1.81	0.3
PPC	Phosphoenolpyruvate carboxylase	0.36	0.13
ME	Malic enzyme	0.15	0.01
ICL	Isocitrate lyase	0	0
AKGDH	2-Oxogluterate dehydrogenase	0.02	0
SUCOAS	Succinyl-CoA synthetase (ADP-forming)	0.02	0
