# Supplementary material for: CRISPR interference screens reveal growth–robustness tradeoffs in Synechocystis sp. PCC 6803 across growth conditions
Source: Plant Cell. 2023 Jul 26;35(11):3937–56. doi: 10.1093/plcell/koad208 (PMC10615215; doi:10.1093/plcell/koad208)
Supplement: koad208_Supplementary_Data [file tpc23.00192Peer Review Report.pdf › tpc23.00192Peer Review Report.pdf]

CRISPR interference screens reveal growth–robustness tradeoffs in *Synechocystis* sp. PCC 6803 across growth conditions

Rui Miao, Michael Jahn, Kiyan Shabestary, Elton Paul Hudson

Corresponding author: Elton P. Hudson (paul.hudson@scilifelab.se)

Review timeline:

**TPC2023-LSB-00192**	Submission received:	February 23, 2023
	1st Decision:	April 11, 2023 *revision requested*
**TPC2023-LSB-00192R1**	1st Revision received:	May 26, 2023
	2nd Decision:	June 26, 2023 *acceptance pending, sent to science editor*
	Final acceptance:	July 20, 2023

REPORT: (The report shows the major requests for revision and author responses. Minor comments for revision and miscellaneous correspondence are not included. The original format may not be reflected in this compilation, but the reviewer comments and author responses are not edited, except to correct minor typographical or spelling errors that could be a source of ambiguity.)

TPC2023-LSB-00192 1st Editorial decision – *revision requested* April 11, 2023

We have received reviews of your manuscript entitled "CRISPR interference screens reveal tradeoffs between growth rate and robustness in *Synechocystis* sp. PCC 6803 across trophic condition." Thank you for submitting your best work to The Plant Cell. The editorial board agrees that the work you describe is substantive, falls within the scope of the journal, and may become acceptable for publication, pending revision and potential re-review.

The three reviewers are supportive of this article and raise a number of issues to improve the manuscript which should be addressed on a point-by-point basis. A main strength of this work relates to the resource that this work represents for the community. As such, the request for a more comprehensive 'lookup' table as suggested by Reviewer 2 would be especially valuable. Amongst other issues raised, the request for a discussion/analysis of the relative RNA levels in representative CRISPRi lines by Reviewer 3 specifically needs to be considered.

Please contact us if there are ambiguous comments or if you wish to discuss the revision.

Given the nature of the comments, we are offering you 60 days from when we have issued this decision to complete the revision. If a revision is not returned within this time frame, and if you have not been granted an extension, we will withdraw the manuscript, which will leave you free to submit the work elsewhere. If you need an extension, we encourage you to contact us at any point before the 60 days have passed.

-- Reviewer comments:

TPC2023-LSB-00192R1 1st Revision received May 26, 2023

Editor comments and **author responses**:

The three reviewers are supportive of this article and raise a number of issues to improve the manuscript which should be addressed on a point-by-point basis. A main strength of this work relates to the resource that this work represents for the community. As such, the request for a more comprehensive 'lookup' table as suggested by Reviewer 2 would be especially valuable. Amongst other issues raised, the request for a discussion/analysis of the relative RNA levels in representative CRISPRi lines by Reviewer 3 specifically needs to be considered.

> **Author's response: We thank the editors' comments and several more comprehensive 'lookup' tables, including all the sgRNA sequences and fitness scores for all the genes, are added to Supplementary Data Set 1 and 2. We also discussed the relative RNA levels in the CRISPRi library in the Discussion section.**

Reviewer comments and **author responses**:

<u>Reviewer #1:</u>

The manuscript by Miao et al describes the creation of a pooled CRISPR interference library in *Synechocystis* PCC 6803 (S6803) to identify mutants with varying fitness effects when tested under 11 different light conditions and carbon sources. The primary goal was to identify genes that are potentially beneficial under one set of conditions but have a fitness cost in others. This would help disentangle where these two major inputs (light and carbon) interact and where they act independently. The second more obvious goal is to identify novel genes that have significant impact on fitness. Since ~45% of genes in S6803 which is the best studied model cyanobacterium still remain unknown, this is a useful aim.
In summary, the results described are often corroborations of what is already established in the field, especially since they chose to look at two inputs light and carbon) that are the most well-studied in terms of physiology. Some findings of lesser general interest are carefully described (e.g. role of the Gap1/2 in glycolysis) and will be of interest to a specialist audience. Several hypotheses are proposed to explain the results they report(e.g. why repression of the major light harvesting antennae, phycobilisomes or PBS actually improves growth under certain conditions). However, without further experiments it is not possible to distinguish between these possibilities and reduce the impact of the report. A recurring theme in this manuscript is that reasonable hypotheses are proposed for the data/results presented but often there are no follow up experiments (e.g. line 214-216; line 15-159; line 330-334; line 610) which is a drawback . However, in a few cases, further evidence is provided by the creation of specific mutants to corroborate hypotheses and this is a strength of the manuscript.

Author's response: We thank the reviewer for the overall positive evaluation and insightful comments. We see this study as a resource for the community that generates many new hypotheses, including hypotheses or findings that are not mentioned explicitly, or were not yet discovered in the data. Given the scope of our study, which involves a comprehensive gene knockdown screen encompassing thousands of genes across 11 different conditions, it is indeed challenging to conduct experimental validations for all hypotheses. However, we did make efforts to validate several selected examples where there is controversial evidence in the literature (role of *gap1*, *gap2*), or where a recently described regulator can be implicated with new functions (*cp12*). We hope that other hypotheses can and will be validated independently in the future by groups that have interest and expertise for specific genes/pathways.

Pooled CRISPRi mutant libraries can reveal novel behaviors and generate powerful hypotheses, in a high throughput manner, which has to be followed up by creation of specific mutants or other confirmatory tests. One of the advances described in the manuscript is the use of ~5 sg RNAs for each gene, rather than the 2 used in the previous study from the same group (a total of ~21000 sgRNAs). This builds the ability to use statistical tools and is useful to the wider community who could use it to address other questions. Another advance is that several small RNAs were also targeted in this study. It is now known that small RNAs are a widespread class in cyanobacteria; unfortunately, the results presented suggest that the effects of mutants were small or negligible.
The webapp is mentioned and is possibly useful as a teaching tool or by researchers to generate other hypotheses. It is not described much in the manuscript. In general, such technologies have not been extensively available to the research community working on cyanobacteria. It is mentioned that the mutant library is frozen and available to other groups, which is definitely an advantage. Thus, this manuscript describes a useful resource and provides several examples of what can be achieved by such a resource (as compared to single gene knockouts that are laborious to create and can sometimes be lethal).

Author's response: We thank the reviewer for the encouragement. We wish to make our results accessible to the community. According to the suggestion, we added more details about the app in the results section, page 6, lines 162-166.

Transformed cells were grown in turbidostats for up to 10 generations (I was not quite sure how many hours this represents) and change in abundance was measured and used to determine a fitness score under 11 different growth conditions that included low, high and fluctuating light as well as 2 different CO_2 levels and conditions for mixotrophy, photoheterotrophy and N starvation. The design and choice of conditions are standard and straightforward, and methods are explained adequately. The results (based on dropouts) showed that pathways associated with protein synthesis and AA biosynthesis had a negative effect on fitness while genes involved in the process of photosynthesis, biosynthesis of cofactors and secondary metabolites including chlorophyll, and oxidative phosphorylation also showed significant adverse effects. They also determined that under certain conditions (low and intermediate light as well as fluctuating light), the fitness penalty for repression of ribosomal proteins was low. They concluded that since under these high stress conditions, growth was reduced, the demand for ribosomes was also reduced. This is an interesting and obvious hypothesis but requires follow up experiments. Without this, the impact of the results is weakened. Pertinent literature from other systems (e.g. from E.coli where there is a great deal of experimental literature on growth rates and protein synthesis DOI: 10.1016/j.cels.2021.06.002) would have been helpful in the discussion. A second finding was that the effect of central carbon metabolism and glycolysis had a much less profound effect than repression of ribosomes etc. on growth. The authors attribute this to three different possibilities, but these are not tested further. Another general conclusion was that when cells grow slower they are relatively

less sensitive to repression of ribosomes. Again, this is a plausible explanation, but unsatisfying because it was not tested in any other way.

RESPONSE: Author's response: We agree with the reviewer that the reduced fitness penalty for ribosomes in the LC, IL and LC, FL conditions is puzzling. Ribosomes are essential for growth in all conditions, and growth was also a prerequisite for all our cultivations as it is needed for mutant selection. However, previous reports have shown that, at very low growth rates a larger fraction of ribosomes become inactive, instead of reducing elongation rate as previously thought (Dai et al., Nat Micro, 2016, PMID: 27941827). In our study, the generation times for these two conditions are 50 h and 69 h, respectively (updated Table 1). We therefore assumed that cells in these conditions have a "reserve" of inactive ribosomes. According to this hypothesis, repression of ribosome biosynthesis affects these cells less than it affects faster growing cells because the buffer of inactive ribosomes can be recruited to maintain growth. This hypothesis is in line with a previous study of the *Synechocystis* protein economy, where we found that slow growing cells did not reduce ribosomes as much as the reduction of growth rate would suggest (Jahn et al., Cell Reports, 2018, PMID: 30304686). Figure 3 D (see below) from that paper shows incomplete reduction of ribosomes (sector "RIB") with decreasing light and CO_2 availability. In addition, a polysome profiling experiment in *Synechocystis* showed that 100S particles (inactive form) increased when cells were in the LC, IL condition compared to the HC, IL condition (Karlsen et al., 2018). This pool of inactive ribosomes may serve as a buffer and become a source of active ribosomes during CRISPRi repression. To fully prove this hypothesis, one would need to determine the fraction of active ribosomes and the effect of CRISPRi repression on ribosome content and activity, something that goes much beyond the scope of this manuscript. To make this reasoning more clear, a brief explanation was added to the respective results section, page 8, lines 190-198.

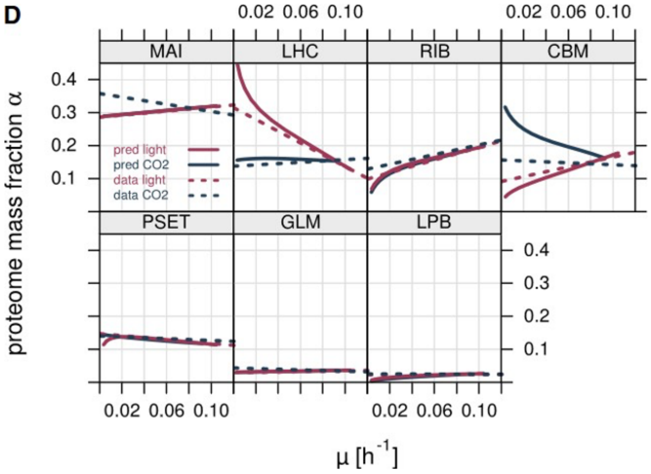

Another major question addressed was if there are genes that are specifically required under conditions where there are changes in light regimes or carbon metabolism pathways. Multiple linear regression analysis was used to distinguish between the specific effect of these inputs (I cannot really comment on this methodology of distinguishing between regimes, although it seems reasonable). They found many proteins that were specific to each of these categories: for example, PBSs and flv proteins in fluctuating light and a proposed regulator ssl3364 (CP12). In follow up experiments, specific knockouts of CP12 were generated and had phenotypes similar to the CRISPRi repression clones. This helps strengthen their argument that there is fine tuning of glycolysis and gluconeogenesis, under light or dark conditions. I cannot really comment on this methodology of distinguishing between regimes, although it seems reasonable. The repression of PBS, had a negative effect except under HL and photoheterotrophy , where repression improved growth rate, from which authors concluded that when this large protein complex is not needed, repression is helpful and improves growth rate.

The next section is devoted to an analysis of fitness tradeoffs in anticipation of light stress and C limitation. Not surprisingly, repression of major subunits of PSI and PSII had a strong negative effect on growth. However, under conditions of photoheterotrophic growth, repression of PSII had a growth advantage, from which authors conclude that the generally high levels of PS II expression inflict a burden on the cells. To further explore the idea that preparation for changes in light intensity can cause cells to have suboptimal growth, under certain conditions, they examined levels of PBS components. This complex makes up a significant part of the proteome, so it is not surprising that under conditions when it is not required

as much, its repression actually helps growth rates. At a general level, this explanation is acceptable, but the lack of detail or follow up detracts from the impact of the manuscript.

Author's response: In recent years, there has been a growing recognition of the concept of a 'protein economy', which asserts that cellular protein resources are finite and need to be balanced to optimize growth or survival. We have investigated this concept in *Synechocystis* and the litho-autotroph *Cupriavidus necator*, and generally found that autotrophs synthesize important protein complexes based on their needs, while maintaining considerable reserves of un-utilized proteins (Jahn et al., Cell Reports, 2018, PMID: 30304686; Jahn et al., eLife, 2021, PMID: 34723797). In the linked studies we also found evidence that reduction of underutilized proteins improves growth, as have others found too for the examples of Rubisco (Makino et al., 1997, PMID: 12223722) and phycobilisomes (Lea-Smith et al., Plant Phys, 2014, PMID: 24760817). More generally, all proteins have an optimal amount according to their flux capacity, and changing the expression level away from the optimum is associated with high fitness costs (Price and Arkin, Genome Bio and Evo, 2016, PMID: 27289091). We therefore think that this explanation is highly plausible, but full experimental proof would go beyond the scope of this study.

A second example of tradeoffs provided, is based on the effect of Flv proteins on growth under fluctuating light. Flv proteins are required for protection under excess light energy. As expected, repression of two of these genes affected cell growth. The explanation of the effect of two other Flv genes was more complicated because they were specific to two conditions. The authors suggest two possible explanations but were unable to distinguish between them: One suggestion was an upregulation of other compensatory genes, or that resources are less utilized allowing for higher growth. These are plausible reasons but there are no follow up experiments to distinguish between these very different explanations.

Author's response: We agree with the reviewer that the phenotype of Flv2/4 repressing strains in LC, IL and LC, LL, +FL conditions are complicated. It has been previously reported that the Flv proteins play a role in alleviating electron pressure downstream of PSI through a Mehler-like reaction under constant light. However, the precise functional mechanism of these proteins requires further investigation, which goes beyond the scope of our current study. In order to address this concern and enhance the clarity of our hypothesis, we have made revisions to the Flv2/4 section in both the results and discussion sections, see pages 12-13, lines 284-319 and page 27, lines 629-636. Our hypothesis, in summary, suggests that the repression of Flv2 (or the Flv2/4 operon) in these specific conditions saves protein resources, while the upregulation of NDH-1_3 and Flv3 is sufficient to dissipate excess electron flow. To address the reviewer's comment, we generated the knockout mutant ΔFlv2 and conducted additional photosynthetic measurements. Our results are in line with a previous study (Figure 4 in Helma et al., 2003,PMID: 12573219) that the deletion of Flv2 did not harm cell photosynthesis capacity. A new figure was added to the Supplementary Data as Figure S4. Although our new results do not fully elucidate the underlying mechanisms driving the observed phenotype, they serve as valuable indications for other research groups interested in conducting more comprehensive investigations on these two proteins in the future.

Minor points and potential for improvements:

Story with the sll0218 was somewhat inconsistent. It is initially described as a gene of unknown function (line 264) but the reference cited does attribute a role. The explanation of the potential role of sll0218 could be more carefully described.

Author's response: We agree, and have changed the respective paragraph. As pointed out by the reviewer, sll0218 is attributed with a function in photosystem repair and stability, but the function seems unrelated to the action of the downstream and upstream Flv genes *sll0217* and *sll0219*.

Another section is devoted to understanding the role of various transporters. I found this quite confusing and I was unable to really decide what was most significant. This section needs to be rewritten with more clarity.

Author's response: We have changed this section significantly to improve clarity, see page 13-15, lines 320-354.

Fitness effect of ncRNA: There are almost 1900 noncoding RNAs. Of these 85% are associated with antisense RNAS; 15 % are small RNAs and of these 27 small RNAs had a significant effect on growth in at least one growth condition. Surprisingly , not much is known about the role of this class of RNAS, so, in general, the detailed analysis which was well-described is important. They conclude that only 2 sRNAS had significant effect, but in most cases, the confounding consequence of polarity effects could not be ruled out.

I was not sure why dCas9 was inserted into psbA1 a choice that was not explained)..mutants in the other two copies had little effect. The explanation is that they compensate for each other. These three proteins have been extensively studied and there is published literature on this. Can they use this to say any more about the roles of the three psbA proteins via this analysis?

Author's response: Integration of dCas9 into the psbA1 locus was based on a report that transcript was only observed from this locus (Mohamed and Jansson 1989 PMID: 2518835) in extremely light (1500 µmmol m-2 s- 1) condition. This report led

to its designation as "neutral site" suitable for transgene integration (Varman et al., AEM, 2013, PMID: 23183979). The psbA1 gene appears to encode a D1 variant D1' that is more specific to low oxygen conditions (Summerfield et al., 2008 PMID: 18998707; Crawford et al 2016 PMID: 26991994). Therefore, psbA1 was not the result of a rigorous comparison of all suitable integration sites. We have revised the corresponding sentence, page 10, lines 239-245. The two remaining psbA copies, psbA2 and psbA3, have been reported to compensate for one another's repression (Mohamed et al., 1993 PMID: 8479422). Our result is further evidence to support this theory since no significant fitness change was observed in any of the tested conditions, which means these two genes sufficiently compensate each other.

I was surprised that the Nbl proteins were not discussed. These small proteins are critical for PBS degradation and could well play a very critical role under the light conditions tested. Were they missed in creation of the sgRNA library since they are so small? This is also a problem that is of general interest in discussing the best strategies for creation of sgRNA libraries.

Author's response: We thank the reviewer for the suggestion. In fact, the NblA1 and NblA2 genes were also targeted by the CRISPRi library. However, we found no significant change in fitness for the two genes in any condition, see the attached heat map for all conditions.

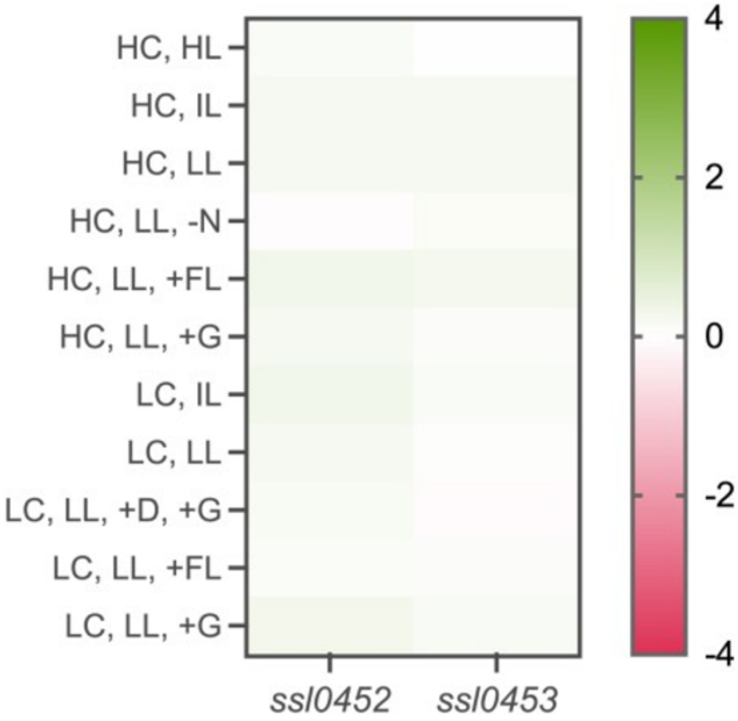

The last section on the prediction of sgRNA from sequence information, although useful, did not seem to be a good fit with the rest of the manuscript and would be better suited to a supplementary section.

Author's response: While we agree that the last section does not present a biological but rather a technical result, the design principles of guide RNAs are of high relevance for the cyanobacterial community. As we had no previous reference of what is a good or bad guide RNA in cyanobacteria, the current library was designed by randomly choosing guides based only on off-target binding and distance to the promoter. With our new results, G content in the seed region, crisproff score, and other features can be included to improve on-target efficiency. We therefore deem these results useful for other groups and matching the scope of our paper as a resource article. In order to provide better access to the guide RNAs we used in this study, we include an additional supplementary material with all the sgRNA sequences and corresponding fitness score in all tested conditions.

Figure 2B: would be helpful to point out the various components of the PBS rods so that the data on the left pane can be linked to the structure.

Author's response: We have added the PBS protein names to figure 2B.

The results section often has information/comments and more general remarks that belong better in the discussion. Eg 392-394: This is more of a recommendation and not a result. In general. the discussion is pretty long and in places confusing. It can be tightened by use of subheadings and used to highlight areas where pooled Crispr mutant libraries are useful and where improvements are required. For example: what is the effect of having all mutants in a turbidostat; are there confounding effects and how can they be ruled out? Other sections could focus on the results where they mainly corroborate published results and others where they lead to new insights.

Author's response: We thank the reviewer for the constructive suggestion. We have added subtitles and rewrote major parts of the discussion section to improve clarity and also added a short discussion about technical limitations.

I am not able to comment on whether the use of specific statistical packages and pipelines is appropriate and should be addressed by an expert.

Figure 2B: Would be helpful to label the subunits of the PBS. It will make it much easier to relate that to the panel showing the data on the subunits (eg apcABCDEF etc.)

Author's response: We have added the PBS protein names to figure 2B, and all the genes are color coded to the corresponding subunit of PBS.

Supplemental figure legends: In many cases, the last two lines were unreadable. I am not sure if that is specific to my output file.

Author's response: We are sorry that the reviewer was not able to read the whole figure legends in the supplemental data file. It may be due to some software/technical problems. High resolution single images will be submitted with the revised version of this paper.

Reviewer #2:

In the manuscript by Miao et al., authors describe their exhaustive analysis of nearly all genes and regulatory regions in the model cyanobacterium, *Synechocystis*, through the use of CRISPR interference-mediated suppression. Impressively, the study includes 11 different cultivation conditions in which the library of suppressor strains (with most genomic targets having 5 different targeting sequences) are evaluated in a pooled population for their fitness relative to other CRISPRi lines. The authors highlight a number of interesting mutants with context-dependent fitness deficits, including many expected mutations based on prior literature and a number of novel/surprising results for certain genes and potential regulatory RNAs. Finally, the work indicates some generalizable design principles for guide RNA that are predictive of high efficiency repression of genetic targets through CRISPRi approaches. In total, the work is exceptional in its comprehensive, systematic examination of cyanobacterial genes under an array of laboratory culture conditions and in culture conditions likely more representative of those encountered in natural environments. It is this reviewer's opinion that this work is of very high quality and is likely to be impactful and important to the fields involving both fundamental and applied research in cyanobacteria. The suggestions and comments below are provided in the spirit of constructive feedback that may be useful for the authors in poising their work for maximal impact and should not be regarded as requirements.

Author's response: We thank the reviewer for the positive and encouraging evaluation on our manuscript.

Minor comments:

The comment that this reviewer most wishes to emphasize is that the work presented in this manuscript is of broad utility to the cyanobacterial community and that the impact of the publication is likely to be directly influenced by the accessibility of the full datasets for the genes collected. Currently, the supplementary materials provide tables for the top gene targets that led to significant fitness suppressing/enhancing effects in each growth condition. This is a useful lookup table for the general 'takeaways' discussed in the manuscript and is appreciated. However, the data on these tables is a small fraction of the data collected - the manuscript is likely to be a more useful community resource (and be cited more) if interested researchers can look up their gene(s) of interest for the conditions and see the fitness impacts, even if not above the threshold cut offs determined. For instance, even "negative results" of little/no fitness impacts may be informative for some genes under certain conditions.

Author's response: We fully agree with this comment. In order to make results more accessible, we have A) added Supplementary data set 1 which contains a full list of all the sgRNA sequences, fitness scores from each sgRNA, and a complete list of all fitness scores; B) added Supplementary data set 2 where we filtered the fitness scores based on their adjusted p-value; C) added a paragraph with more details about the Shiny App that contains this data (Results, page 6, line 162-166). The app allows all interested researchers to browse the data set and visualize fitness for genes of interest without the need to work with downloaded tables.

Perhaps the most unexpected result of the CRISPRi is that there is such a strong fitness enhancement in the Flv2/4 downregulated lines under some conditions that also contain low inorganic carbon (including fluctuating light). The authors suggest that this might be due to protein burden and it is certainly possible that protein synthesis is partially to blame. However the magnitude of the fitness enhancement appears to be on the extreme tail relative to almost all other significant genes (i.e., in the range of a +5 fitness score). This reviewer suggests that protein synthesis burden is unlikely to account for such a dramatic fitness change. Perhaps the authors have some other hypotheses, even if they are speculative, that might be briefly discussed?

Author's response: The fitness effect observed for Flv2-4 was indeed higher than what can be expected simply from the protein burden that the two proteins constitute, and comparably high to some of Syenchocystis' most strongly expressed genes (phycobilisome subunits). We suspect that a mixture of effects might be at play, including but not limited to protein burden. As what we answered to a related question from Reviewer 1, our hypothesis, in summary, suggests that the repression of Flv2 (or the Flv2/4 operon) in these specific conditions saves protein resources, while the upregulation of NDH-13 and Flv3 is sufficient to dissipate excess electron flow.

Less important, but related to the above: the suppression of PSII under DCMU conditions is likely to reduce not only the burden of protein synthesis (line 219) but also to reduce the formation of deleterious charge recombination from the blocked Qb site (and subsequent ROS formation).

Author's response: We thank the reviewer for the suggestion and have included it in the manuscript, page 10, lines 245-254. Indeed, adding DCMU to light-exposed cells may result in elevated ROS accumulation at PSII since electrons cannot be transferred further downstream of the linear electron transport chain. Then repressing PSII reduces the chance of this ROS elevation. However, it is hard to estimate to what extent it helped the growth of corresponding mutants under photoheterotrophic condition, since these mutants still showed significant fitness decrease in excess light due to the essential role of PSII there.

The authors may wish to have a brief discussion of classes of suppressed genes that might be expected to have a different phenotype in a mixed population of pooled mutants rather than in axenic cultures of the same background. For example, suppression of important metabolic pathways where other cells in the mixed culture may supply 'rescuing' metabolites. Or other phenotypes the authors have discussed that might be less (or more) pronounced in this type of mixed analysis.

Author's response: We thank the reviewer for this interesting and inspiring thought. We have, so far, always treated the individual strains as quasi-independent. This is of course a simplification. Two cases can be considered where cross talk between the population and the knock-down strain exists: A gain-of- function or a loss-of-function mutant. In the first case, a mutant might suddenly secrete a compound that is either toxic, beneficial, or growth-modulating for other strains (quorum sensing), affecting fitness of the whole population. We deem this effect negligible as long as all strains are relatively evenly distributed in the population. If for example only 1 in 20,000 bacteria secretes a toxin or quorum sensing hormone, the concentration will be too low to affect other cells. The second case is more relevant: A strain loses the capability to synthesize a certain metabolite but it is provided by the rest of the population. In this case the repression mutant has no fitness defect and the essentiality of the gene remains undiscovered. We have added a new paragraph about limitations of pooled library experiments to the discussion.

Figure 1B: Please define "FC" in the legend and/or main text prior to its use in this figure. Additionally, the X-axis on these graphs is likely mislabeled (I believe the authors mean to state "generations" rather than hours). Finally, it is not explicitly stated how the generation time was calculated for each of the different conditions and/or what the difference between generation times were for the 11 environmental alterations.

Author's response: Thanks for this comment. It was indeed a mislabeling on the X-axis on Figure 1B, we have corrected it to 'generations'. We also added an additional column in Table 1 to show all generation times of the library for all 11 cultivation conditions. The 'FC' in Figure 1B was spelled out as 'fold change'.

Figure 2A-D: In these proofs, the figure was a little fuzzy/low resolution. This made some things like the determination of the stars indicating significance a bit difficult to view when printed at full size.

Figure 4A: Consider if these red/green colors are differentiable for color blind individuals and if a different color scheme could improve accessibility.

Author's response: We have made changes to the scaling of the bar charts that hopefully improve visibility of significance symbols. The color palette used throughout the manuscript is in fact a colorblind-friendly palette named 'Dark2' from R colorbrewer, see for example https://www.datanovia.com/en/blog/the-a-z-of- rcolorbrewer-palette/. We have also verified with an app that the colors are distinguishable for the most common types of color blindness.

Figure 4DE: The genomic context for these highlighted ncRNAs is less useful at a glance when the gene names aren't provided (or perhaps these genes encode mostly genes of unknown function). Crispoff score is referenced multiple times in the manuscript but is only defined quite late in the main text (line 699). Similar comment for 'one-hot encoding'.

Author's response: The gene names in Figure 4 were added according to the suggestion. The definitions for 'Crispoff' score and 'one-hot-encoding' were added to the first appearance of the terms.

Reviewer #3:

This manuscript presents an in-depth, genome-wide examination of the fitness effects of the ORFs and ncRNAs in the cyanobacterium *Synechocystis* sp. PCC 6803 under a wide range of environmental conditions. This is achieved through CRISPRi, which involves the use of the dCas9 system. Both the mutant generation and range of conditions examined represent an expansion of those used by this group in an earlier publication (Yao et al. 2020. Nat Comm 11: 1666). This is a well conducted analysis that provides interesting new insights regarding the fitness tradeoffs of many gene products in varying light and nutrient conditions.

Author's response: We thank the reviewer for the positive evaluation on our manuscript.

General Comments:

Although the authors are relying on CRISPRi to generate the phenotypes they examine, there is no data presented that quantitatively show the extent to which RNA levels actually decrease in the various mutants because of this approach, only relative changes in fitness in different locations within genes (Fig 1C). This may have been established in a previous publication. If so, it should be discussed, since a 100% decrease in RNA levels from any given gene because of CRISPRi will be interpreted much differently than if it is a lower percentage. The multiple copies of the 6803 chromosome further complicate this issue. This issue is mentioned briefly but not really addressed in the Discussion.

Author's response: We draw from a series of reports where the transcript abundance of CRISPRi targets in *Synechocystis* was measured using a similar genetic construct (both dCas9 and sgRNA expressed from PL22 promoter). From these examples, median repression was 88% (n=18, s.d. 9.7%). There was no trend between gene expression level before repression and repression efficiency (i.e. highly expressed genes are also repressed 90%). We have now added this statistic to the manuscript, page 28, lines 665-671, and provide a new table (Table S2) in Supplementary Data.

Specific Comments

Table 1 and Fig 1F legend. Many abbreviations are used here, and although some of these are provided in the text, I had to really search for many of them. I suggest writing out the conditions and providing the abbreviations in Table 1, and where needed, the same in the legend for Fig 1F.

Author's response: We agree that there are many similar abbreviations for the conditions. To make it easier to

follow, we have written out the conditions in Table 1.

Table 1. Do you mean light intensity or irradiance? I suspect the latter, but you have the former. Also, change "mmol per meter squared per second" to "micromol photons per meter squared per second".

Author's response: Thank you for the suggestion, the error was corrected on the unit. However, we kept the usage of light intensity since it is a commonly used term in the field.

Fig 1F. The writing on the x-axis is often incomplete and their meaning is not immediately obvious. Perhaps the writing can be angled a bit more, and more words used when appropriate, along with abbreviations used when they are obvious.

Author's response: The x-axis labels in Figure 1F were improved according to the suggestion.

Results. The Results section should be tightened up considerably. It contains a significant amount of background information and many interpretations of the results throughout it. These should be presented either in the Introduction or in the Discussion. Only the information needed to contextualize the presentation of the results should be provided in this section.

Author's response: We thank the reviewer for the suggestion and we have tightened the result section and rewrote the major part of the discussion section.

Line 236. Replace "phycobilisomes" with "PBS".

Author's response: We have replaced "phycobilisomes" with "PBS" in several places after the abbreviation is introduced in the manuscript.

Fig. 2C and 2D legends. Provide descriptions of the illustrations on the right, as was provided for Figs. 2A and 2B.

Author's response: Figure legend was modified accordingly.

Fig 3A. The lines connecting the fitness charts to the central diagram are too light in color and need to be darkened considerably.

Author's response: The changes to the figure were made accordingly.

Fig 4 legend. Change "...effect on fitness." to "...effect on fitness for the conditions tested."

Author's response: The changes to the figure were made accordingly.

TPC2023-LSB-00192R1 2nd Editorial decision – *acceptance pending* June 26, 2023

We are pleased to inform you that your paper entitled "CRISPR interference screens reveal tradeoffs between growth rate and robustness in *Synechocystis* sp. PCC 6803 across trophic condition" has been accepted for publication in The Plant Cell, pending a final editorial review by a science editor. At this stage, your manuscript will be evaluated by a science editor with respect to its presentation of scientific content, compliance with journal policies, and presentation for a broad readership.

Final acceptance from Science Editor July 20, 2023
